# Supplementary material for: Structure of papain-like protease from SARS-CoV-2 and its complexes with non-covalent inhibitors
Source: Nat Commun. 2021 Feb 2;12:743. doi: 10.1038/s41467-021-21060-3 (PMC7854729; doi:10.1038/s41467-021-21060-3)
Supplement: Supplementary file 1 — Supplementary Information [file 41467_2021_21060_MOESM1_ESM.pdf]

Supplementary Information

Supplementary Table 1. Data processing and refinement statistics.

Structure	PLpro WT 100K 1.79 Å	PLpro C111S mutant 100K 1.60 Å	PLpro C111S mutant RT 2.50 Å	PLpro C111S mutant – 1 100K, 2.09 Å	PLpro C111S mutant – 2 100K , 1.95 Å	PLpro C111S mutant – 3 100K, 2.05 Å	PLpro WT – 3 100K, 2.30 Å
**Data processing**							
Space group	$P3_221$	$P3_221$	$P3_221$	$I4_122$	$I4_122$	$I4_122$	$I4_122$
Cell dimensions<br>*a*=*b*, *c* (Å)<br>*α*=*β*, *γ* (°)	 82, 134<br>90, 120	 82, 135<br>90, 120	 82, 135<br>90, 120	 114, 220<br>90, 90	 114, 219<br>90, 90	 114, 220<br>90, 90	 114, 220<br>90, 90
Resolution range (Å)[a]	1.79 (1.84–1.79)	1.60 (1.64-1.60)	2.50 (2.54-2.50)	2.09 (2.13-2.09)	1.95 (1.98-195)	2.05 (2.09-2.05)	2.3 (2.34-2.30)
Unique reflections [a]	49,598 (2,456)	69,708 (3,105)	19,357 (942)	42,950 (2,097)	52,219 (2,368)	45,458 (2,245)	32,308 (1,576)
R-merge[b]	0.137 (1.78)	0.095 (0.871)	0.167 (1.61)	0.145 (1.94)	0.097 (1.23)	0.101 (1.71)	0.095 (1.73)
Mean I/sigma(I)	27.2 (1.72)	34.8 (1.71)	17.2 (1.4)	21.0 (1.07)	35.2 (1.08)	42.4 (1.03)	44.3 (0.97)
CC1/2[c]	0.991 (0.632)	0.996 (0.741)	0.987 (0.514)	0.995 (0.512)	1.00 (0.559)	1.00 (0.566)	1.01 (0.605)
Completeness (%)	100 (100)	99.1 (89.5)	100 (100)	99.8 (99.3)	99.5 (91.6)	99.9 (99.6)	99.9 (99.0)
Redundancy	14.2 (12.4)	13.7 (8.3)	9.8 (8.9)	12.6 (11.5)	16.6 (9.5)	21.0 (15.2)	20.9 (14.6)
Wilson B-factor ($Å^2$)	23.0	33.6	54.4	51.8	36.3	49.2	66.3
**Refinement**							
Resolution range (Å)	48.8-1.79	49.0-1.60	49.3-2.50	49.7-2.09	49.7-1.95	45.4-2.05	45.5-2.30
Reflections work/test	47,056 (2,498)	66,140 (3,516)	18,320 (1,004)	40,785 (2,153)	49.570 (2,620)	43,228 (2,194)	30,715 (1,564)
R_{work}/R_{free}	0.160 / 0.174	0.123 / 0.164	0.151 / 0.193	0.186 / 0.300	0.175 / 0.190	0.188 / 0.201	0.212 / 0.239
Number atoms							

Protein	2575	2599	2539	2495	2561	2496	2426
Ligand / ion	25	30	7	48	57	51	47
Water	223	380	41	127	210	135	32
Protein residues	318	318	318	318	318	318	318
RMSD (bonds) (Å)	0.010	0.009	0.010	0.010	0.011	0.010	0.006
RMSD (angles) (°)	1.49	1.43	1.58	1.62	1.65	1.68	1.49
Rotamer outliers (%)[d]	1.38	0.34	2.47	1.82	1.40	2.18	1.50
Clashscore[d]	2.71	1.72	2.18	2.20	2.7	3.59	2.27
Average B-factor (Å2)	39.3	31.4	59.7	67.7	49.1	66.0	89.9
Protein	38.6	29.6	59.8	68.1	48.8	66.3	90.2
Ligand / ion	57.9	41.1	63.2	72.3	50.5	67.5	85.9
Water	45.1	42.6	50.0	59.6	51.4	59.1	71.7
Number of TLS groups	1	1	1	1	1	1	1
**PDB ID**	6WZU	6WRH	6XG3	7JIR	7JIT	7JIV	7JIW

[a] Values in parentheses correspond to the highest resolution shell.
[b] Rmerge = $\Sigma h \Sigma j|Ihj - \langle Ih \rangle|/\Sigma h \Sigma j Ihj$, where Ihj is the intensity of observation j of reflection h.
[c] As defined by Karplus and Diederichs [1].
[d] As defined by Molprobity

Supplementary Table 2. Sequence of PLpro synthetic gene used for expression of protein in *E. coli* and list of primers for cloning PLpro gene into pMCSG53 plasmid and generation of C111S mutant.

Sequence of synthetic gene for PLpro

GAAGTTCGTACCATCAAAGTTTTCACCACCGTTGACAACATCAACCTGCACACCCAGGTTGTTGACATGT
CCATGACCTACGGTCAGCAGTTCGGTCCGACCTACCTGGATGGTGCTGATGTTACCAAAATTAAACCGCA
CAACTCTCACGAAGGTAAAACCTTCTACGTTCTGCCGAACGATGACACCCTGCGTGTTGAAGCGTTCGAA
TACTACCACACCACCGATCCGTCTTTCCTGGGTCGTTATATGAGCGCTCTGAACCACACCAAAAAATGGA
AATACCCGCAGGTTAACGGTCTCACCTCTATCAAATGGGCTGATAACAACTGCTACCTGGCGACCGCGCT
GCTGACCCTGCAGCAGATCGAACTGAAATTCAACCCGCCGGCACTGCAGGATGCTTACTACCGTGCTCGT
GCTGGTGAAGCGGCTAACTTCTGTGCGCTGATCCTGGCTTATTGCAACAAAACCGTTGGTGAACTGGGTG
ATGTTCGTGAAACCATGTCTTACCTGTTCCAGCACGCTAACCTGGACTCCTGTAAACGTGTACTGAACGT
TGTTTGTAAAACCTGCGGTCAGCAGCAGACCACCCTGAAAGGTGTTGAAGCTGTTATGTACATGGGCACC
CTGAGCTACGAACAGTTCAAAAAAGGCGTTCAGATCCCGTGTACCTGCGGTAAACAGGCGACCAAATACC
TGGTTCAGCAGGAATCTCCGTTCGTTATGATGTCTGCTCCGCCGGCGCAGTATGAACTGAAACACGGCAC
CTTCACCTGCGCGTCTGAATATACCGGTAACTACCAGTGCGGTCACTACAAACACATCACCTCTAAAGAA
ACCCTGTACTGCATCGATAAAGGCCCGATCACCGACGTTTTCTACAAAGAAAACTCTTACACCACCACCA
TTAAATAA

Primers for cloning PLpro gene into PMCSG53 plasmid

Nsp3_pMCSG53_F_	TACTTCCAATCCAATGCCGAAGTTCGTACCATCAAAGTTTTCACCA
Nsp3_pMCSG53_R_	TTATCCACTTCCAATGTTATTTAATGGTGGTGGTGTAAGAGTTTTCTTTGTA

Primers for cloning C111S mutant

PLpro,C111S-F	CTGATAACAACAGCTACCTGGCGACCGCGCT
PLpro,C111S-R	CCAGGTAGCTGTTGTTATCAGCCCATTTGATAGAGGTGA

Supplementary Methods

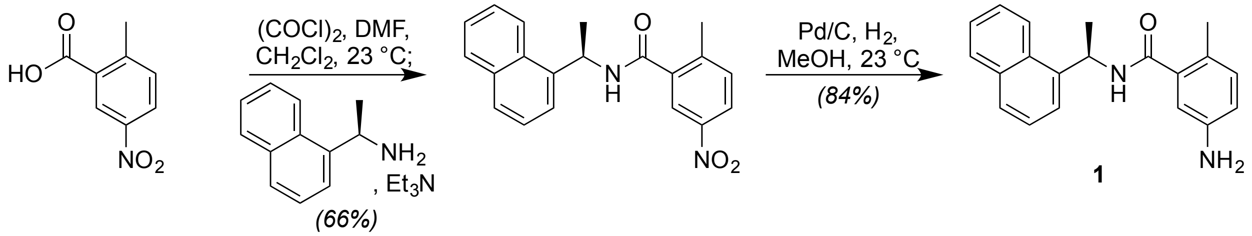

Synthesis of Compound 1 (GRL0617)

To a suspension of the 5-nitro-*o*-toluic acid (0.362 g, 2.00 mmol, 1.0 equiv) in CH_2Cl_2 (10.0 mL) at 0 °C was added $(COCl)_2$ (0.206 mL, 2.40 mmol, 1.2 equiv) dropwise before catalytic DMF (8 drops from a 1.00 mL syringe) was added dropwise. This mixture was then stirred at 0 °C for 30 min before being concentrated directly. The resultant residue was then redissolved in CH_2Cl_2 (20.0 mL) at 23 °C before the sequential addition of (*R*)-(+)-1-(1-naphthyl)ethylamine (0.417 mL, 2.60 mmol, 1.3 equiv) and Et_3N (0.558 mL, 4.00 mmol, 2.0 equiv). The reaction mixture was then stirred at 23 °C for 30 min. Upon completion, the reaction contents were quenched by the addition of 1 M HCl (30 mL), diluted with CH_2Cl_2 (10 mL), and poured into a separatory funnel. The two phases were separated, and the organic layer was washed with 1 M NaOH (3 × 30 mL). The organic extract was then dried (Na_2SO_4), filtered, and concentrated. Purification of the resultant residue by flash column chromatography (silica gel, hexanes:EtOAc, 2:1) afforded the desired amide intermediate (0.440 g, 66% yield) as a white solid. Pd/C (0.200 g, 10% by weight, 0.19 mmol, 0.15 equiv) was then carefully added to a suspension of the newly formed amide intermediate (0.430 g, 1.29 mmol, 1.0 equiv) in MeOH (30.0 mL) at 23 °C. The resultant suspension was then purged by direct bubbling with a balloon of H_2 gas for 2 h at 23 °C. Upon completion, the reaction contents were filtered through a short pad of Celite, washed with EtOAc (200 mL), and dried (Na_2SO_4) to directly provide inhibitor **1** (0.328 g, 84% yield) as a white solid. **1 (GRL0617)**: R_f = 0.20 (silica gel, hexanes:EtOAc, 1:1); IR (film) ν_{max} 3339, 3049, 2976, 2927, 1639, 1511, 1339, 1244, 1121, 817, 800, 755 cm^{-1}; ^1H NMR (400 MHz, $CDCl_3$) δ 8.23 (d, *J* = 8.7 Hz, 1 H), 7.88 (d, *J* = 8.9 Hz, 1 H), 7.81 (d, *J* = 8.1 Hz, 1 H), 7.60–7.49 (m, 3 H), 7.46 (dd, *J* = 8.2, 7.2 Hz, 1 H), 6.93 (d, *J* = 7.9 Hz, 1 H), 6.60–6.54 (m, 2 H), 6.16–6.07 (m, 1 H), 5.97 (d, *J* =

8.5 Hz, 1 H), 3.51 (s, 2 H), 2.29 (s, 3 H), 1.77 (d, *J* = 6.7 Hz, 3 H); ^{13}C NMR (101 MHz, $CDCl_3$) δ 169.2, 144.2, 138.2, 137.1, 134.1, 131.9, 131.3, 128.9, 128.6, 126.7, 126.1, 125.5, 125.3, 123.7, 122.7, 116.8, 113.5, 44.9, 20.8, 18.9; $[\alpha]_D^{22}$ = –75.8° (*c* = 1.0, $CHCl_3$) [lit. $[\alpha]_D^{20}$ = –76.8° (*c* = 1.0, $CHCl_3$) from *J. Med. Chem.* **2009**, *52*, 5228].

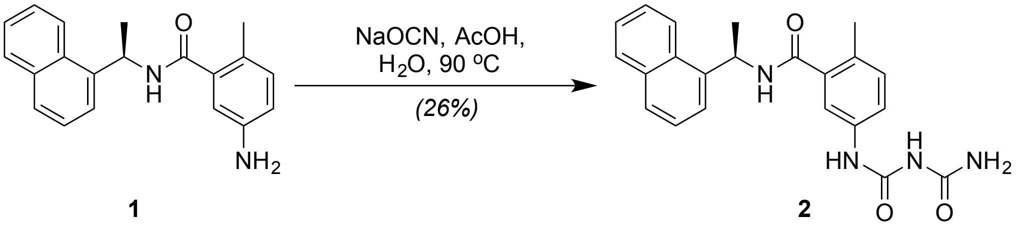

1 **2**

Synthesis of Compound 2

To a solution of amine **1** (30.0 mg, 0.10 mmol, 1.0 equiv) in CH_3CN/H_2O (1:1) (1.2 mL) was added NaOCN (51.3 mg, 0.80 mmol, 8.0 equiv) and the mixture was heated at 90 °C for 15 min. Then, AcOH (23.2 μL, 24.2 mg, 0.40 mmol, 4.0 equiv) was added dropwise, and the stirring was continued for additional 1 h at 90 °C. Then, the second portion of AcOH (23.2 μL, 24.2 mg, 0.40 mmol, 4.0 equiv) was introduced, and the resulting solution was stirred for 3 h at 90 °C. Upon completion, the mixture was cooled to 23 °C, the precipitate filtered, washed with H_2O (5 × 2 mL) and dried (using air) to afford compound **2** (10.6 mg, 26% yield) as a white solid. **2**: R_f = 0.20 (silica gel, EtOAc). IR (film) ν_{max} 3284, 2975, 2928, 1704, 1640, 1548, 1496, 1408, 1228, 1201, 801, 779 cm^{-1}; ^{1}H NMR (400 MHz, DMSO-*d*$_6$) δ 9.97 (s, 1H), 8.90 (d, *J* = 8.0 Hz, 1 H), 8.86–8.84 (m, 1 H), 8.24 (d, *J* = 8.4 Hz, 1 H), 7.98–7.92 (m, 1 H), 7.87–7.80 (m, 1 H), 7.63–7.49 (m, 4 H), 7.43–7.35 (m, 2 H), 7.15 (d, *J* = 8.2 Hz, 1 H), 7.05–6.71 (m, 2 H), 5.91 (p, *J* = 7.1 Hz, 1 H), 2.22 (s, 3 H), 1.57 (d, *J* = 6.9 Hz, 3 H); ^{13}C NMR (101 MHz, DMSO-*d*$_6$): δ 167.8, 155.5, 152.0, 140.2, 137.6, 135.6, 133.4, 130.8, 130.4, 129.5, 128.7, 127.3, 126.2, 125.6, 125.5, 123.2, 122.5, 119.8, 117.7, 44.3, 21.5, 18.6; HRMS (ESI) calculated for $C_{22}H_{23}N_4O_3^+$ [M + H$^+$] 391.1765, found 391.1761; $[\alpha]_D^{22}$ = –102.3° (*c* = 0.2, acetone).

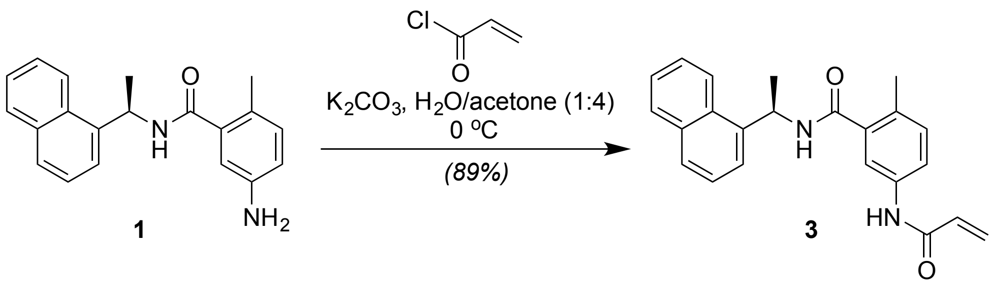

Synthesis of Compound 3

To a solution of K_2CO_3 (18.2 mg, 0.132 mmol, 2.0 equiv) in H_2O (0.12 mL) and acetone (0.48 mL) at 0 °C was added acryloyl chloride (0.011 mL, 0.132 mmol, 2.0 equiv). Amine **1** (20.0 mg, 0.066 mmol, 1.0 equiv) was then added dropwise at 0 °C as a solution in acetone (0.2 mL) and the reaction mixture was allowed to stir at 0 °C for 10 min. Upon completion, the reaction contents were quenched by the addition of saturated aqueous NH_4Cl (10 mL), diluted with CH_2Cl_2 (10 mL), and poured into a separatory funnel. The two phases were separated, and the aqueous layer was extracted with CH_2Cl_2 (3 × 10 mL). The combined organic extracts were then dried (Na_2SO_4), filtered, and concentrated. Purification of the resultant residue by flash column chromatography (silica gel, hexanes:EtOAc, 1:1) afforded the desired acrylamide **3** (21.0 mg, 89% yield) as a white solid. **3**: R_f = 0.27 (silica gel, hexanes:EtOAc, 1:1); IR (film) ν_{max} 3276, 3052, 2977, 1707, 1611, 1596, 1541, 1496, 1411, 1244, 1202, 982, 799, 778 cm^{-1}; ^1H NMR (400 MHz, $CDCl_3$) δ 8.19 (d, *J* = 8.3 Hz, 1 H), 8.06 (s, 1 H), 7.85 (dd, *J* = 7.9, 1.7 Hz, 1 H), 7.77 (d, *J* = 8.2 Hz, 1 H), 7.56–7.45 (m, 3 H), 7.44–7.37 (m, 2 H), 7.32 (s, 1 H), 6.99 (d, *J* = 8.3 Hz, 1 H), 6.43 (d, *J* = 8.3 Hz, 1 H), 6.34 (dd, *J* = 16.9, 1.5 Hz, 1 H), 6.19 (dd, *J* = 16.9, 10.1 Hz, 1 H), 6.12–6.02 (m, 1 H), 5.66 (dd, *J* = 10.1, 1.5 Hz, 1 H), 2.29 (s, 3 H), 1.73 (d, *J* = 6.8 Hz, 3 H); ^{13}C NMR (110 MHz, $CDCl_3$) δ 169.0, 163.9, 138.2, 136.5, 135.5, 134.1, 132.1, 131.6, 131.2, 131.1, 129.0, 128.6, 127.9, 126.7, 126.0, 125.4, 123.5, 122.8, 122.0, 118.8, 45.2, 21.0, 19.3; HRMS (ESI) calculated for $C_{23}H_{23}N_2O_2^+$ [M + H^+] 359.1754, found 359.1754; $[\alpha]_D^{22}$ = –110.7° (*c* = 1.0, acetone). [Note: a slight concentration dependence was observed for NMR spectra of this compound].

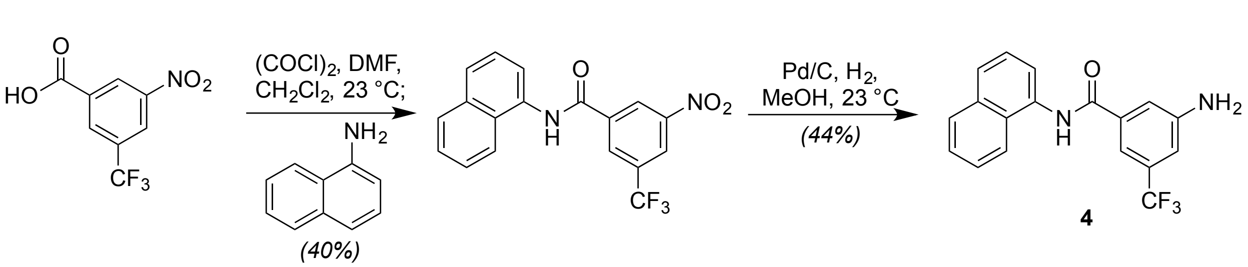

Synthesis of Compound 4

3-nitro-5-(trifluoromethyl)benzoic acid (0.752 g, 3.20 mmol, 1.0 equiv) was suspended in CH_2Cl_2 (14 mL) under an argon atmosphere at 23 °C. Then, DMF (2 drops) was added, followed by slow addition of oxalyl chloride (0.300 mL, 0.444 g, 3.50 mmol, 1.1 equiv). The resulting solution was stirred at 23 °C for 1 h before complete dissolution of the starting material was observed. The mixture was then concentrated to dryness, back-filled with argon, redissolved in CH_2Cl_2 (10 mL) and cooled to 0 °C. Then, naphthylamine (0.500 g, 3.49 mmol, 1.1 equiv) was added in a single portion and the solution was warmed to 23 °C and stirred vigorously at this temperature for 2 h. The resulting precipitate was filtered and washed with cold CH_2Cl_2 (2 × 10 mL). The CH_2Cl_2-containing filtrate was discarded, and the filter cake was then thoroughly washed with warm (50 °C) EtOAc (4 × 20 mL). The filtrate was concentrated to afford the desired amide (0.460 g, 40% yield) as a white solid. R_f = 0.36 (silica gel, hexanes:EtOAc = 5:1); ^{1}H NMR (400 MHz, DMSO-d_6) δ 11.00 (s, 1 H), 9.19–9.12 (m, 1 H), 8.88 (s, 1 H), 8.78–8.73 (m, 1 H), 8.09–7.99 (m, 2 H), 7.96–7.87 (m, 1 H), 7.69–7.53 (m, 4 H). Next, to a suspension of the newly formed amide (0.050 g, 0.14 mmol, 1.0 equiv) in MeOH/EtOAc (2 mL, 1:1) at 23 °C was flushed several times with nitrogen and then charged with Pd/C (10 wt %, 20 mg). After flushing the resulting solution several times with H_2, the reactions contents were left to stir at 23 °C under a H_2 atmosphere for 12 h. Upon completion, the resulting solution was filtered through Celite® (washing with MeOH) and concentrated. The resulting crude product was suspended in CH_2Cl_2 (2 mL) followed by addition of methanol (with stirring) until a clear solution was obtained. The resulting mixture was placed in the freezer (–20 °C) overnight. The precipitate was then collected by filtration, rinsed with CH_2Cl_2 and dried on high vacuum to afford compound **4** (20.0 mg, 44% yield) as a white solid. **4**: R_f = 0.30 (silica gel, hexanes:EtOAc, 1:1); IR (film) ν_{max} 3355, 3229, 3053, 1627, 1605, 1526, 1504, 1371, 1264, 1168, 1122, 998, 867, 691 cm^{-1}; ^{1}H NMR (400 MHz, DMSO-d_6) δ 10.47 (s, 1 H), 8.03–7.92 (m, 2 H), 7.91–7.84 (m, 1 H), 7.61–7.45 (m, 6 H), 7.11–7.00 (m, 1 H), 5.93–5.82 (br s, 2 H); ^{13}C NMR (101 MHz, DMSO-d_6) δ 165.7, 149.8, 136.3, 133.8, 129.9 (q, J = 31.3 Hz), 129.2, 128.1, 126.4, 126.1, 126.0, 125.7, 125.6, 124.0, 123.3, 123.0, 116.7, 112.1 (q, J = 4.0 Hz), 110.6 (q, J = 3.9 Hz); ^{19}F NMR (470 MHz, DMSO-d_6) δ –61.34; HRMS (ESI) calculated for $C_{18}H_{14}F_3N_2O^+$ [M + H$^+$] 331.1053, found 331.1052.

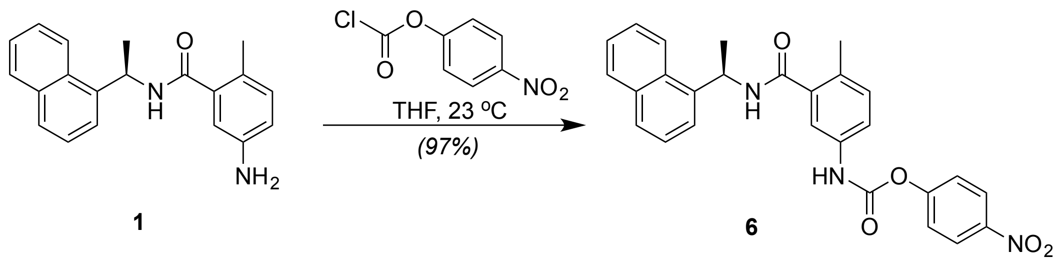

Synthesis of Compound 6

To a solution of amine **1** (21.9 mg, 0.072 mmol, 1.0 equiv) in THF (0.70 mL) at 23 °C was added 4-nitrophenyl chloroformate (21.8 mg, 0.108 mmol, 1.5 equiv) and the reaction mixture was allowed to stir at 23 °C for 1 h. Upon completion, the solution was concentrated directly. Purification of the resultant residue by flash column chromatography (silica gel, hexanes:EtOAc, 2:1) afforded compound **6** (32.4 mg, 97% yield) as a white solid. **6**: R_f = 0.27 (silica gel, hexanes:EtOAc, 1:1); IR (film) ν_{max} 3254, 3051, 2051, 1735, 1638, 1603, 1523, 1489, 1345, 1201, 1011, 858, 778 cm^{-1}; ^1H NMR (500 MHz, $CDCl_3$) δ 8.19–8.16 (m, 1 H), 8.15–8.12 (m, 2 H), 7.88–7.83 (m, 1 H), 7.80–7.77 (m, 1 H), 7.77–7.74 (m, 1 H), 7.54–7.46 (m, 3 H), 7.45–7.38 (m, 2 H), 7.35–7.29 (m, 1 H), 7.20–7.14 (m, 2 H), 7.09 (d, *J* = 8.3 Hz, 1 H), 6.26–6.21 (m, 1 H), 6.10 (p, *J* = 6.8 Hz, 1 H), 2.35 (s, 3 H), 1.74 (d, *J* = 6.8 Hz, 3 H). ^{13}C NMR (126 MHz, $CDCl_3$) δ 168.7, 155.3, 150.5, 148.4, 145.0, 137.8, 137.0, 134.9, 134.1, 131.8, 131.2, 129.0, 128.7, 126.7, 126.1, 125.3, 125.2, 123.4, 122.7, 122.2, 120.7, 117.7, 45.2, 20.8, 19.2; HRMS (ESI) calculated for $C_{27}H_{24}N_3O_5^+$ [M + H^+] 470.1710, found 470.1703; $[\alpha]_D^{20}$ = –78.4° (*c* = 1.0, $CHCl_3$).

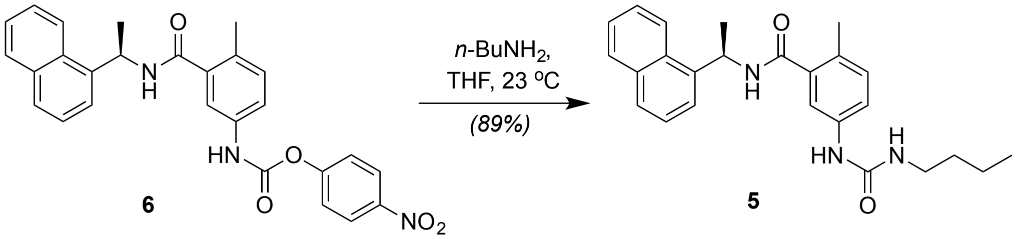

Synthesis of Compound 5

To a solution of **6** (8.5 mg, 0.018 mmol, 1.0 equiv) in THF (0.20 mL) at 23 °C was added *n*-butylamine (0.01 mL, 0.036 mmol, 2.0 equiv) and the reaction mixture was allowed to stir for 5 min. Upon completion, the reaction contents were quenched by the addition of H_2O (5 mL), diluted with CH_2Cl_2 (5 mL), and poured into a separatory funnel. The two phases were separated, and the

aqueous layer was extracted with CH_2Cl_2 (3 × 30 mL). The organic extract was then dried (Na_2SO_4), filtered, and concentrated. Purification of the resultant residue by flash column chromatography (silica gel, CH_2Cl_2:MeOH, 40:1) afforded compound **5** (6.3 mg, 89% yield) as a white solid. **5**: R_f = 0.41 (silica gel, CH_2Cl_2:MeOH, 40:1); IR (film) ν_{max} 3276, 2978, 2946, 2738, 2603, 1633, 1548, 1445, 1236, 1172, 1037, 806 cm^{-1}; ^1H NMR (400 MHz, $CDCl_3$) δ 8.21 (d, *J* = 8.4 Hz, 1 H), 7.88 (d, *J* = 7.7 Hz, 1 H), 7.80 (d, *J* = 8.2 Hz, 1 H), 7.58–7.49 (m, 3 H), 7.45 (dd, *J* = 8.2, 7.2 Hz, 1 H), 7.21–7.15 (m, 2 H), 7.07 (d, *J* = 8.2 Hz, 1 H), 6.25 (s, 1 H), 6.11 (d, *J* = 5.6 Hz, 2 H), 4.66 (s, 1 H), 3.17 (q, *J* = 6.7 Hz, 2 H), 2.34 (s, 3 H), 1.77 (d, *J* = 6.3 Hz, 3 H), 1.47–1.40 (m, 2 H), 1.31 (dd, *J* = 15.0, 7.5 Hz, 2 H), 0.90 (t, *J* = 7.3 Hz, 3 H); ^{13}C NMR (126 MHz, DMSO-d_6) δ 168.2, 155.2, 140.3, 138.1, 137.4, 133.4, 130.5, 128.7, 127.6, 127.2, 127.0, 126.2, 125.6, 125.5, 123.2, 122.5, 118.3, 116.2, 44.2, 38.7, 31.9, 21.5, 19.5, 18.5, 13.7; HRMS (ESI) calculated for $C_{25}H_{30}N_3O_2^+$ [M + H^+] 404.2333, found 404.2337; $[\alpha]_D^{20}$ = –32.5° (*c* = 0.4, acetone).

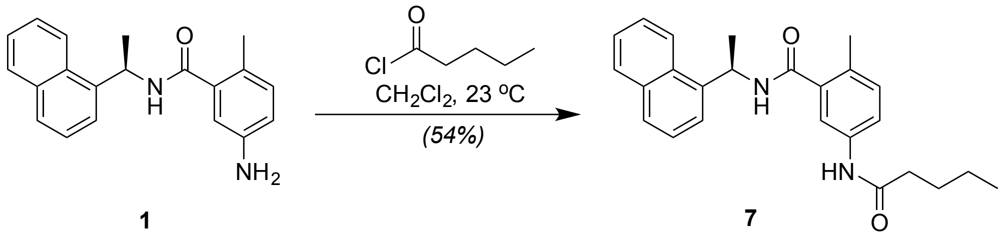

Synthesis of Compound 7

To a solution of amine **1** (24.4 mg, 0.080 mmol, 1.0 equiv) in CH_2Cl_2 (0.800 mL) at 23 °C was added valeroyl chloride (0.010 mL, 0.083 mmol, 1.0 equiv) dropwise. Upon completion, the reaction contents were quenched by the addition of H_2O (1 mL), diluted with CH_2Cl_2 (1 mL), and poured into a separatory funnel. The two phases were separated, and the aqueous layer was extracted with CH_2Cl_2 (3 × 1 mL). The combined organic extracts were then dried (Na_2SO_4), filtered, and concentrated. Purification of the resultant residue by flash column chromatography (silica gel, hexanes:EtOAc, 1:1) afforded compound **7** (16.8 mg, 54% yield) as a colorless oil. **7**: R_f = 0.50 (silica gel, hexanes:EtOAc, 1:1); IR (film) ν_{max} 3274, 3051, 2959, 2929, 2871, 1638, 1594, 1541, 1452, 1338, 1188, 1091, 823, 799, 777 cm^{-1}; ^1H NMR (400 MHz, $CDCl_3$) δ 8.22 (d, *J* = 8.4 Hz, 1 H), 7.87 (d, *J* = 8.0 Hz, 1 H), 7.80 (d, *J* = 8.2 Hz, 1 H), 7.59–7.41 (m, 5 H), 7.37 (d, *J* = 8.2 Hz, 1 H), 7.29 (s, 1 H), 7.08 (d, *J* = 8.2 Hz, 1 H), 6.18 (d, *J* = 8.4 Hz, 1 H), 6.10 (app p, *J* = 6.8 Hz, 1 H), 2.36 (s, 3 H), 2.28 (t, *J* = 7.6 Hz, 2 H), 1.77 (d, *J* = 6.8 Hz, 3 H), 1.65 (p, *J* = 7.6

Hz, 2 H), 1.36 (hex, J = 7.4 Hz, 2 H), 0.92 (t, J = 7.4 Hz, 3 H); ^{13}C NMR (101 MHz, $CDCl_3$) δ 171.7, 168.7, 138.1, 136.8, 135.8, 134.1, 131.9, 131.6, 131.3, 129.0, 128.6, 126.7, 126.1, 125.4, 123.6, 122.8, 121.6, 118.4, 45.1, 37.5, 27.7, 22.5, 20.9, 19.4, 13.9.; HRMS (ESI) calculated for $C_{25}H_{29}N_2O_2^+$ [M + H$^+$] 389.2224, found 389.2220; $[\alpha]_D^{20}$ = –81.7° (c = 0.3, $CHCl_3$).

Synthesis of S1

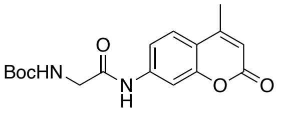

A solution of Boc glycine (507.8 mg, 2.89 mmol, 1.0 equiv), oxyma (0.540 g, 3.79 mmol, 1.2 equiv), DIC (0.538 μL, 3.47 mmol, 1.2 eq) and fluorophore amine (0.389 g, 2.22 mmol, 0.75 eq) in dry DMF (15 mL) was first stirred at 35 °C for 1 h and then overnight at 45 °C. The reaction mixture was concentrated upon reduced pressure and purification by column chromatography (silica gel, 2-10% MeOH:CH_2Cl_2) yielded **S1** in 78% purity as analyzed by LCMS. For further purification, the impure mixture was dissolved in minimal CH_2Cl_2, washed with water and brine. The organic layer was cooled to 0 °C and the white precipitate was filtered to yield pure **S1** (0.562 g, 76% yield). **S1**: ^{1}H NMR (400 MHz, DMSO-d_6) δ 10.39 (s, 1 H), 7.78–7.69 (m, 2 H), 7.48 (dd, J = 8.7, 2.0 Hz, 1 H), 7.12 (t, J = 6.1 Hz, 1 H), 6.26 (d, J = 1.3 Hz, 1 H), 3.77 (d, J = 6.1 Hz, 2 H), 2.40 (d, J = 1.2 Hz, 3 H), 1.40 (s, 9 H); LRMS (ESI) calculated for $C_{17}H_{21}N_2O_5$ [M + H$^+$] 333.14, found 333.4. [2]

Synthesis of S2

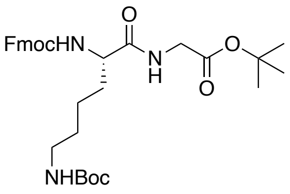

EDC•HCl (5.68 g, 29.5 mmol, 1.3 equiv) was added to a solution of Fmoc-Lys(Boc)-OH (10.02 g, 22.7 mmol, 1.0 equiv), HOBt (4.34 g, 23.8 mmol, 1.05 equiv), HCl•H_2N-Gly-OtBu (3.80 g, 22.7 mmol, 1.0 equiv) and i-Pr_2NEt (5.93 mL, 34.05 mmol, 1.5 equiv) in DMF (50 mL) at 23 °C. The resulting reaction mixture was stirred for 16 h at 23 °C and then concentrated under reduced pressure. Purification by column chromatography (silica gel, 30-70% EtOAc:hexane) yielded **S2** (11.3 g, 86% yield). **S2**: ^{1}H NMR (400 MHz, $CDCl_3$) δ 7.76 (d, J = 7.5 Hz, 2 H), 7.59 (d, J = 7.7 Hz, 2 H), 7.39 (t, J = 7.5 Hz, 2 H), 7.31 (td, J = 7.4, 1.2 Hz, 2 H), 6.53 (s, 1 H), 5.52 (s, 1 H), 4.66 (s, 1 H), 4.40 (d, J = 8.2 Hz, 2 H), 4.21 (t, J = 6.9 Hz, 2 H), 3.92 (s, 2 H), 3.11 (d, J = 5.3 Hz, 2 H), 1.97–1.80 (m, 1 H), 1.60–1.75 (m, 2 H), 1.30–1.55 (m, 21 H); LRMS (ESI) calculated for $C_{32}H_{44}N_3O_7$ [M + H$^+$] 582.31, found 582.6.[3]

Synthesis of S3

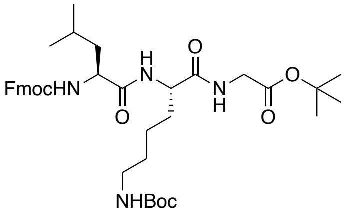

A solution of **S2** (1.392 g, 2.39 mmol, 1.0 equiv) in 10% Piperidine:CH_2Cl_2 (20 mL) was stirred for 20 min at 23 °C and followed with evaporation by rotary evaporation. Dilution with DMF (20 mL) and evaporation by rotary evaporation was repeated two more times to remove residual piperidine. The resultant crude was resuspended in DMF (30 mL), Fmoc leucine (0.928g, 2.62 mmol, 1.1 equiv), HOBt (80%, 0.445 g, 2.63 mmol, 1.2 equiv) and EDC•HCl (0.596 g, 3.10 mmol, 1.3 equiv) were added then added at 23 °C. The reaction mixture was stirred for 30 min at 23 °C and concentrated under reduced pressure. Purification by column chromatography (silica gel, 10-50% EtOAc:CH_2Cl_2) yielded **S3** (1.659 g, 85% yield). **S3**: ^{1}H NMR (400 MHz, $CDCl_3$) δ 7.74 (d, *J* = 7.6 Hz, 2 H), 7.58 (d, *J* = 7.4 Hz, 2 H), 7.38 (t, *J* = 7.5 Hz, 2 H), 7.29 (dd, *J* = 7.5, 3.4 Hz, 2 H), 6.93 (s, 1 H), 6.84 (d, *J* = 7.1 Hz, 1 H), 5.68 (s, 1 H), 4.81 (s, 1 H), 4.62–4.48 (m, 1 H), 4.42 (dd, *J* = 10.6, 7.2 Hz, 1 H), 4.34 (t, *J* = 8.8 Hz, 1 H), 4.25 (d, *J* = 5.1 Hz, 1 H), 4.19 (t, *J* = 7.1 Hz, 1 H), 3.99–3.80 (m, 2 H), 3.03 (d, *J* = 6.6 Hz, 2 H), 2.01 (s, 1 H), 1.87 (h, *J* = 7.6 Hz, 1 H), 1.74–1.51 (m, 4 H), 1.43 (s, 9 H), 1.40 (s, 9 H), 1.38–1.22 (m, 3 H), 0.92 (d, *J* = 6.5 Hz, 6 H); ^{13}C NMR (101 MHz, $CDCl_3$) δ 172.72, 171.60, 168.84, 156.56, 143.96, 143.82, 141.39, 127.84, 127.19, 125.24, 120.10, 82.41, 77.48, 76.84, 67.20, 53.76, 52.89, 47.24, 42.11, 41.52, 32.06, 29.50, 28.56, 28.14, 24.81, 23.13, 22.62, 21.96; HRMS (ESI) calculated for $C_{38}H_{54}N_4O_8$ [M^+] 694.3942, found 694.3945.

Synthesis of S4

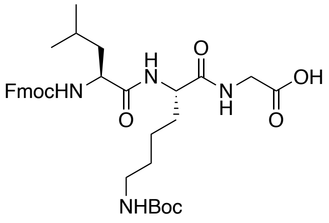

A solution of **S3** (1.23 g, 1.77 mmol, 1.0 equiv) and Et_3SiH (1.4 mL, 8.76 mmol, 5 equiv) in 25% TFA:CH_2Cl_2 (28 mL) was stirred at 23 °C for 18 h, followed by dilution with CH_2Cl_2 (20 mL) and solvent removal by rotary evaporation. Dilution with CH_2Cl_2 (30 mL) and solvent evaporation by rotary evaporation was repeated two more times to remove residual TFA. The resultant crude material was resuspended in MeOH (15 mL) followed by addition of Boc_2O (0.511 g, 2.22 mmol, 1.25 equiv) and *i*-Pr_2NEt (2.29 mL, 13.1 mmol, 7.5 equiv). The reaction mixture was stirred for 1 h at 23 °C, diluted with EtOAc (100 mL), and washed with aqueous HCl (pH ~2). The aqueous layer was further extracted by EtOAc (2 × 30 mL), the combined organic layers were dried over Na_2SO_4, and then evaporated to give a crude product. Purification by column chromatography (silica gel,

3-15% MeOH:CH_2Cl_2) yielded **S4** (0.781 g, 87% purity by LCMS). **S4**: HRMS (ESI) calculated for $C_{34}H_{46}N_4O_8$ [M^+] 638.3316, found 638.3312

Synthesis of S5

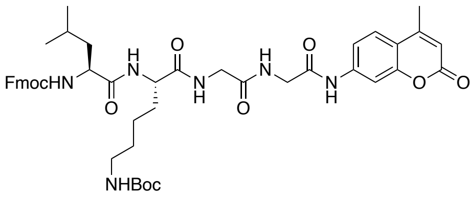

A solution of **S1** (47.8 mg, 0.143 mmol) in 20% TFA:CH_2Cl_2 (2 mL) was stirred at 23 °C for 30 min, followed by dilution with CH_2Cl_2 (10 mL) and solvent removal by rotary evaporation. Dilution with CH_2Cl_2 (15 mL) and solvent removal by rotary evaporation was repeated two more times to remove residual TFA. The resultant crude was resuspended in DMF (5 mL) and deprotected amine intermediate (2.7 mL, 77.4 μmol, 1.0 equiv) was added to a flask containing **S4** (49.7 mg, 77.8 μmol, 1.0 equiv) and *i*-Pr_2NEt (81.0 μL, 0.46 mmol, 6.0 equiv) in DMF (1 mL) at 23 °C. After the addition of EDC•HCl (31.4 mg, 0.15 mmol, 2.0 equiv) the reaction mixture was stirred for 16 h at 23 °C, diluted by EtOAc (20 mL), and then washed with aqueous HCl (pH ~2-3). The aqueous layer was further extracted by EtOAc (2 × 20 mL), the combined organic layers were dried over Na_2SO_4, and then evaporated to give a crude product. Purification by column chromatography (silica gel, 1-6% MeOH in 1:1 EtOAc:CH_2Cl_2) yielded **S5** (22.7 mg, 34% yield). **S5**: ^{1}H NMR (400 MHz, DMSO-d_6) δ 10.15 (s, 1 H), 8.50–8.38 (m, 1 H), 8.26 (t, J = 5.9 Hz, 1 H), 8.07 (d, J = 6.8 Hz, 1 H), 7.88 (d, J = 7.6 Hz, 2 H), 7.79 (d, J = 2.0 Hz, 1 H), 7.75–7.66 (m, 3 H), 7.56–7.47 (m, 2 H), 7.40 (t, J = 6.9 Hz, 2 H), 7.30 (tdd, J = 7.4, 2.2, 1.2 Hz, 2 H), 6.71 (d, J = 6.7 Hz, 1 H), 6.27 (d, J = 1.3 Hz, 1 H), 4.36–4.14 (m, 4 H), 4.13–4.02 (m, 2 H), 3.95 (d, J = 5.9 Hz, 2 H), 3.84–3.71 (m, 2 H), 3.17 (d, J = 5.2 Hz, 1 H), 2.87 (d, J = 5.9 Hz, 2 H), 2.39 (d, J = 1.3 Hz, 3 H), 1.55 (td, J = 41.8, 8.0 Hz, 4 H), 1.34 (s, 12 H), 0.79 (dd, J = 10.8, 6.6 Hz, 6 H); HRMS (ESI) calculated for $C_{46}H_{56}N_6O_{10}Na$ [M + Na^+] 875.3956, found 875.3944.

Synthesis of S6

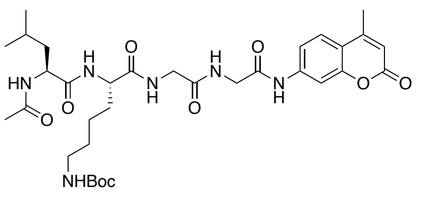

A solution of **S5** (22.7 mg, 26.6 μmol, 1.0 equiv) in 10% Piperidine:CH_2Cl_2 (2 mL) was stirred for 15 min at 23 °C and followed with solvent removal by rotary evaporation. Dilution with DMF (5 mL) and solvent removal by rotary evaporation was repeated two more times to remove residual piperidine. The resultant crude was resuspended in DMF (2 mL), and *i*-Pr_2NEt

(18.5 μL, 0.11 mmol, 4 equiv) and acetic anhydride (10.0 μL, 0.11 mmol, 4 equiv) were added at 23 °C. The reaction mixture was stirred for 1 h at 23 °C, diluted by EtOAc (20 mL), and then washed by aqueous HCl (pH ~2-3). The aqueous layer was further extracted by EtOAc (2 × 20 mL), the combined organic layers were dried over Na_2SO_4, and then evaporated to give a crude product. Purification by column chromatography (silica gel, 2-15% MeOH in 1:1 EtOAc:CH_2Cl_2) yielded **S6** (9.7 mg, 56% yield). **S6**: ^{1}H NMR (400 MHz, DMSO-d_6) δ 10.18 (s, 1 H), 8.39–8.30 (m, 1 H), 8.26 (t, *J* = 5.8 Hz, 1 H), 8.06 (d, *J* = 7.0 Hz, 1 H), 7.98 (d, *J* = 8.1 Hz, 1 H), 7.80 (d, *J* = 2.1 Hz, 1 H), 7.73 (d, *J* = 8.7 Hz, 1 H), 7.54 (dd, *J* = 8.7, 2.1 Hz, 1 H), 6.79–6.68 (m, 1 H), 6.27 (d, *J* = 1.3 Hz, 1 H), 4.29 (q, *J* = 7.6 Hz, 1 H), 4.16 (q, *J* = 6.5 Hz, 1 H), 3.94 (s, 2 H), 3.84–3.67 (m, 2 H), 2.86 (t, *J* = 6.4 Hz, 2 H), 2.40 (d, *J* = 1.3 Hz, 3 H), 1.83 (s, 3 H), 1.73–1.45 (m, 4 H), 1.36 (s, 11 H), 1.23 (s, 3 H), 0.78 (dd, *J* = 6.6, 3.9 Hz, 6 H); HRMS (ESI) calculated for $C_{33}H_{48}N_6O_9$ [M^+] 672.3483, found 672.3502.

Synthesis of CV-2

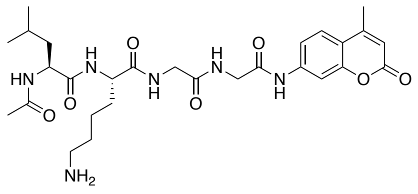

A solution of **S6** (11.2 mg, 16.6 μmol, 1.0 equiv) in 20% TFA:CH_2Cl_2 (2 mL) was stirred at 23 °C for 30 min, followed by dilution with CH_2Cl_2 (5 mL) and solvent removal by rotary evaporation. Dilution with CH_2Cl_2 (5 mL) and solvent removal by rotary evaporation was repeated two more times to remove residual TFA. The crude was dissolved in DMSO to generate 20 mM stock of **CV-2**. Purity was assayed by LC/MS. **CV-2**: HRMS (ESI) calculated for $C_{28}H_{40}N_6O_7$ [M^+] 572.2958, found 572.2956.

Supplementary Data 1 file contains NMR spectra of compounds described in Supplementary Information.

Supplementary Figures

Supplementary Figure 1. Sequence alignment of PLpro homologues. Sequence alignment of SARS-CoV-2 PLpro homologues from CoV-2, SARS, MERS and SADS coronaviruses with structures available in the PDB: SARS CoV-2 (PDB id: 6WZU, this work), SARS CoV (PDB ids: 5Y3E[4] and 3MJ5 [5]), MERS CoV (PDB ids: 5V69[6] and 4RNA[7]) and SADS CoV (PDB id: 6L5T [8]). The secondary-structure elements are labelled for SARS-CoV-2 PLpro.

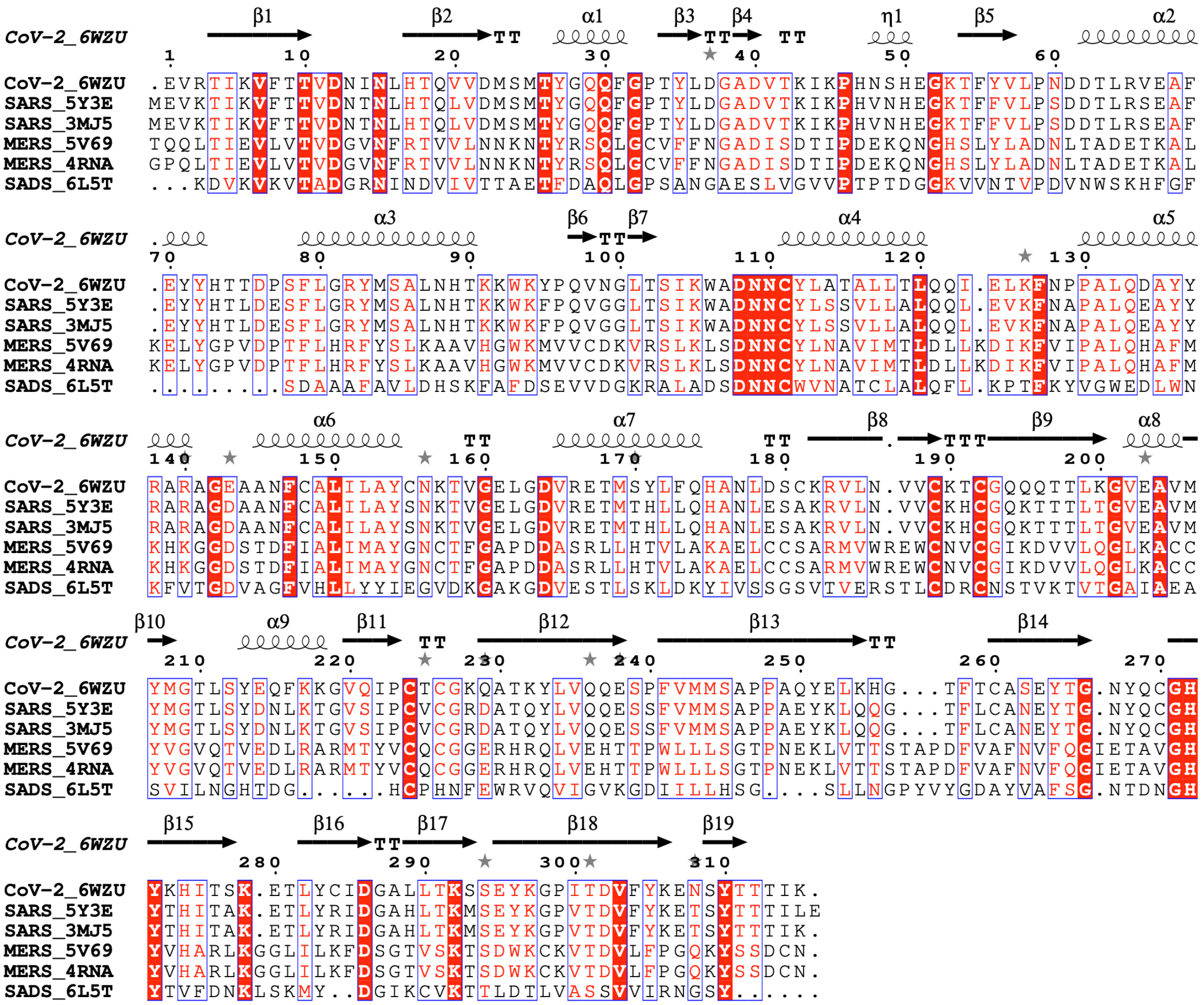

Supplementary Figure 2A. Synthesis of PLpro inhibitors. Synthesis of inhibitors 1-7 starting from commercially available materials.

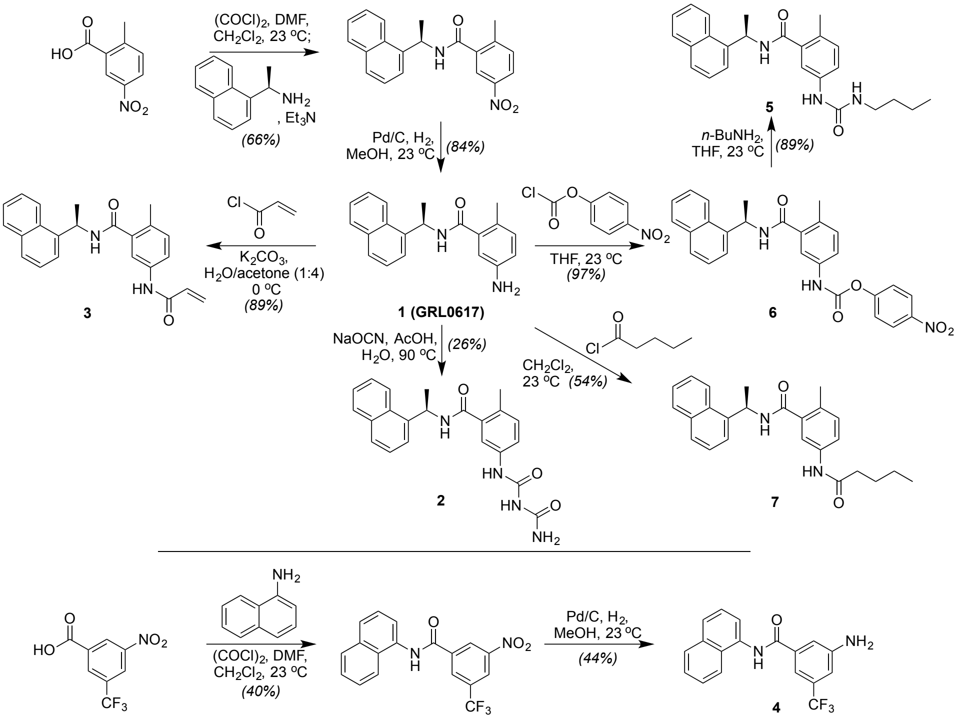

Supplementary Figure 2B. Synthesis of CV-2. Steps in the synthesis of CV-2 compound.

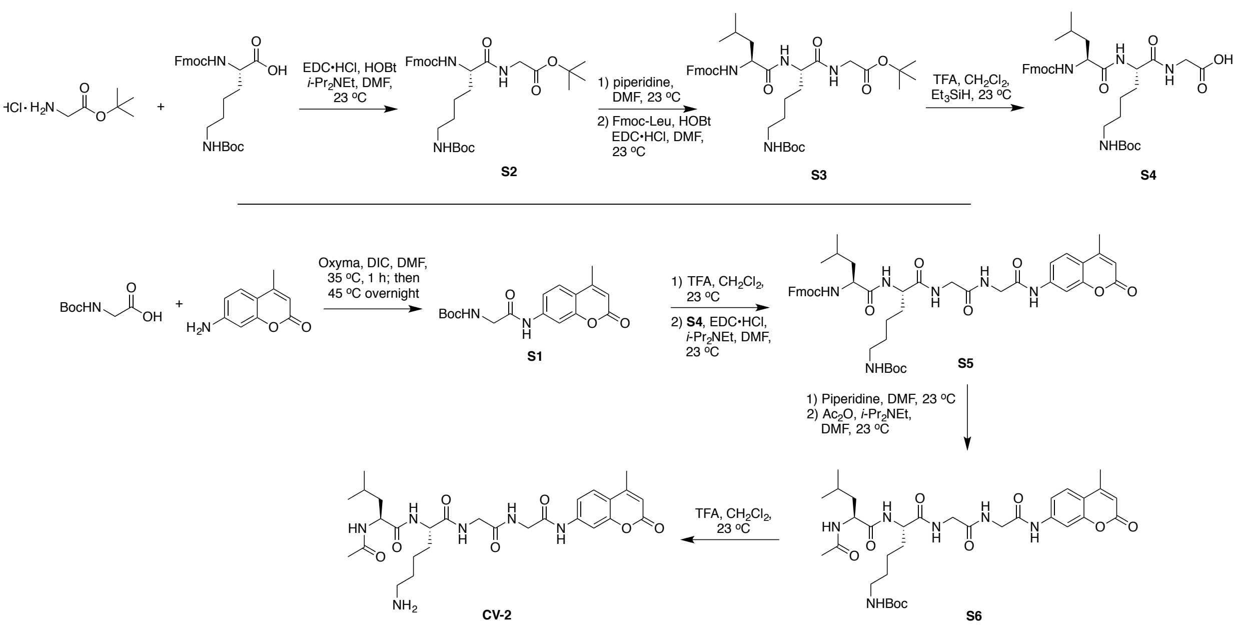

Supplementary Figure 3: Biochemical assay for PLpro. A) CV-2 features a PLpro peptide substrate tethered to a profluorescent molecule which is cleaved when enzymatic activity of PLpro releases a fluorescent product. B) CV-2 (40 μM) incubated with varying amounts of PLpro and fluorescence quantified over time by plate reader. C) Comparison of wild-type to active site mutant (C111S) shows biochemical assays reports on active proteolysis of PLpro. D) PLpro assay performed in the presence and absence of EDTA.

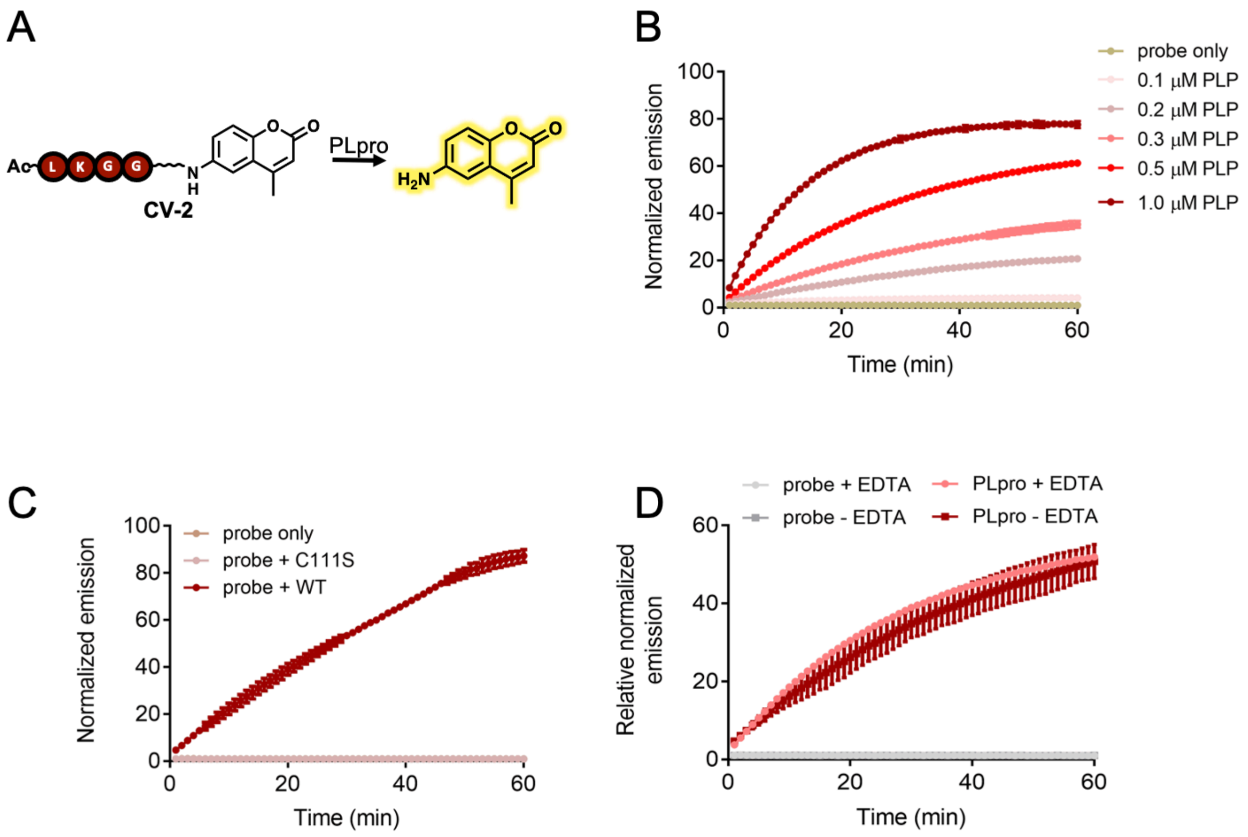

Supplementary Figure 4. Whole cell assay for compound 1. Percent Spike positive cells, n=100 for 3 biological replicates each. Scale bar is 100 μM.

% Positive cells in view (**1**)

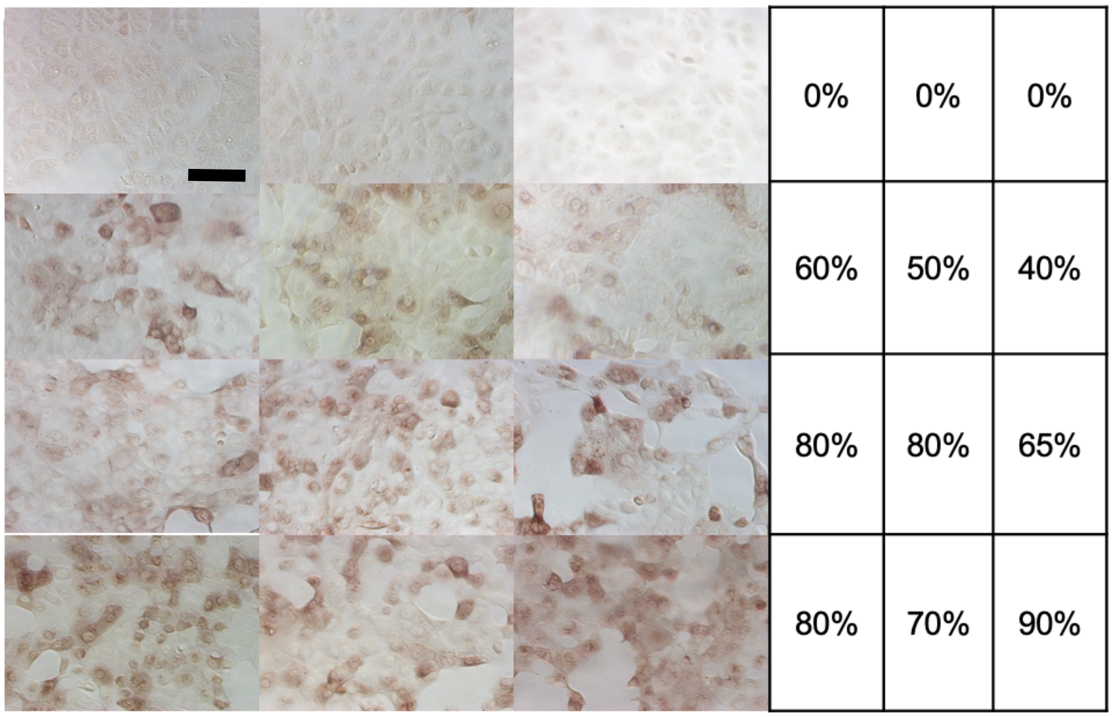

10 mM	0%	0%	0%
1 mM	60%	50%	40%
0.1 mM	80%	80%	65%
0 mM	80%	70%	90%

Supplementary Figure 5. Comparison of PLpro Palm domains. Comparison of Palm domains between SARS Cov2 (magenta) and MERS (dark blue) PLpro. Catalytic triad residues are shown in sticks. Large differences are indicated by black arrows.

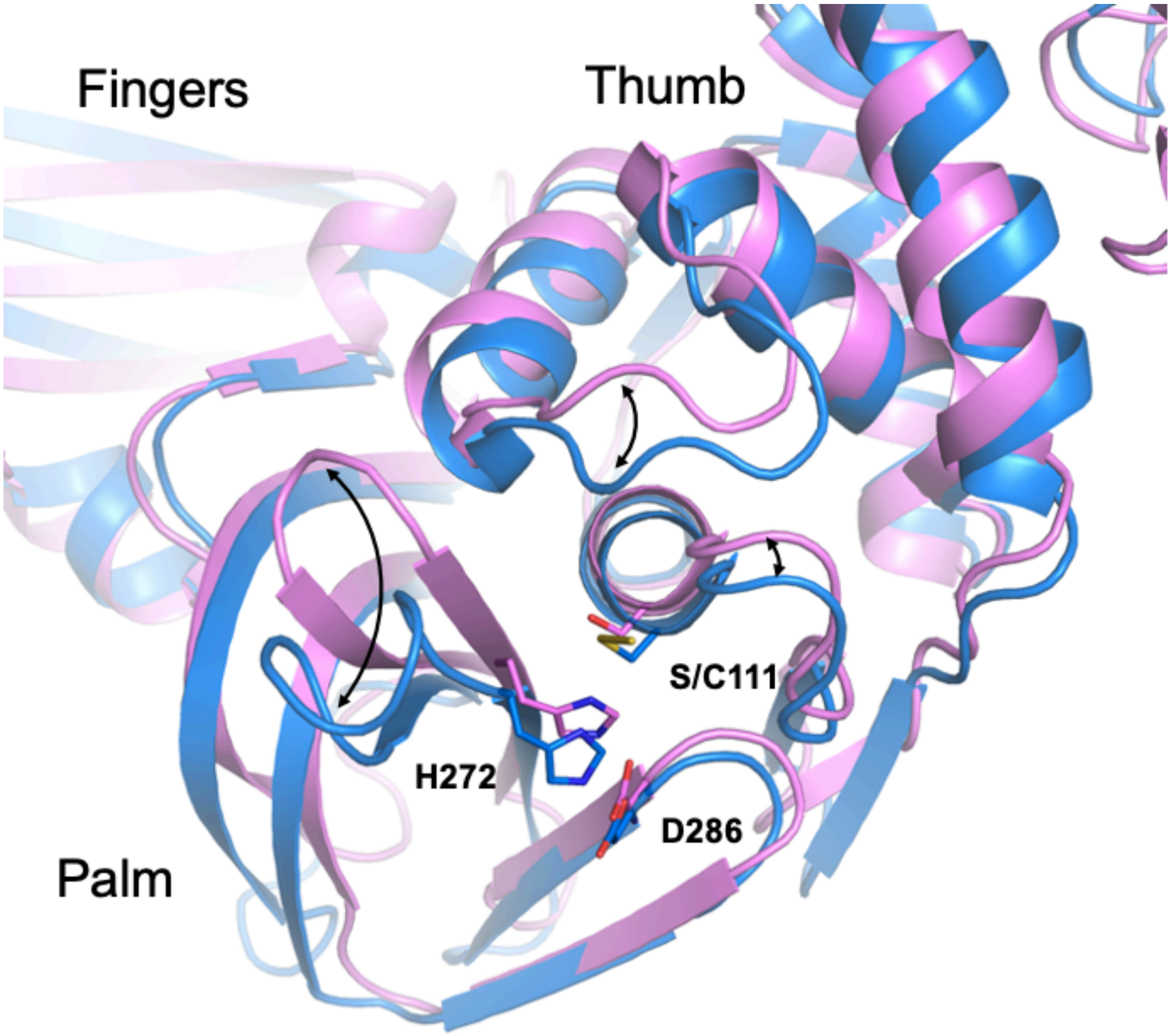

Supplementary Figure 6. SARS-CoV-2 PLpro ligand binding. F_o-F_c electron-density omit maps (green mesh) contoured at 2.2 σ for the ligands (magenta sticks) binding to SARS-CoV-2 PLpro (in magenta). A) Compound **1** binding to C111S PLpro. B) Compound **2** binding to C111S PLpro. C) Compound **3** binding to C111S PLpro. D) Compound **3** binding to WT SARS-CoV-2 PLpro.

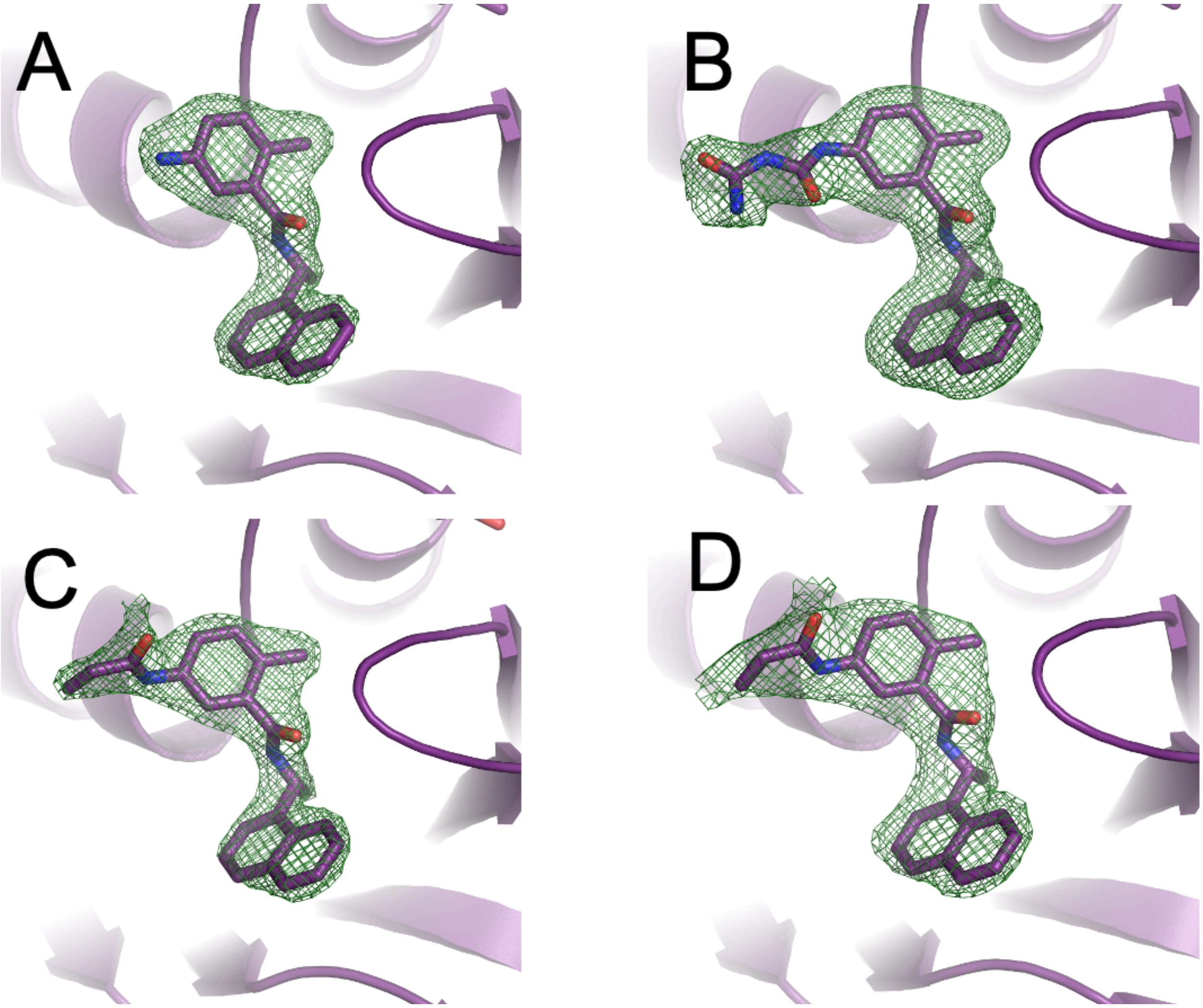

Supplementary Figure 7. PLpro ligand binding.

A) Superposition of PLpro ligand-binding sites of the unliganded WT protein structure (shown in blue, 6WZU id: PDB) and the structure with compound **2** (in magenta with the ligand in green, PDB id: 7JIT).

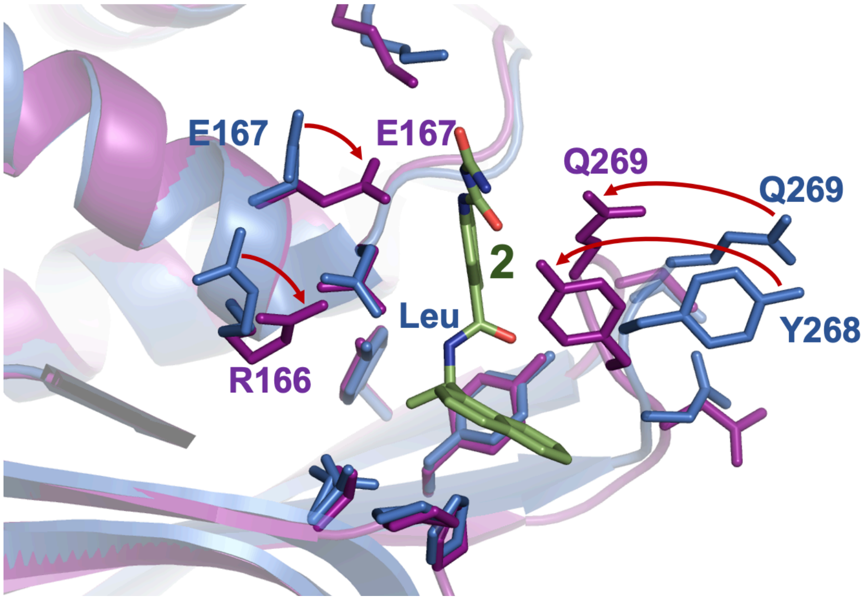

B) Structure superposition of ligand-binding sites of PLpro compound **2** complex (in magenta with ligand in green, PDB id: 7JIT) and SARS-CoV PLpro C112S mutant in complex with ubiquitin (shown in blue with C-terminal part of ubiquitin shown as blue-navy blue sticks, PDB id: 4M0W).

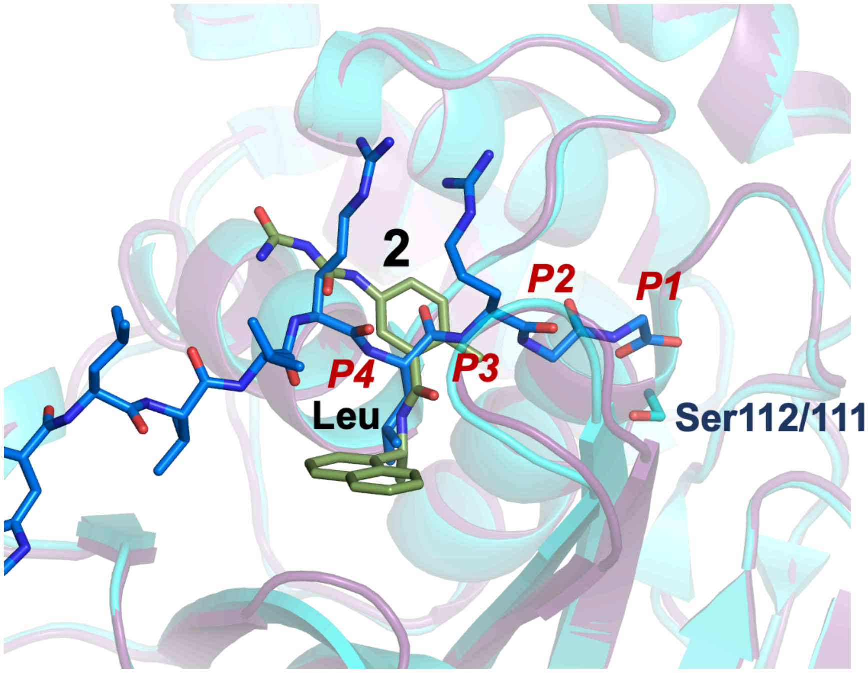

Supplementary References

1 Karplus, P. A. & Diederichs, K. Linking crystallographic model and data quality. *Science* **336**, 1030-1033, doi:10.1126/science.1218231 (2012).

2 Laconde, G., Amblard, M. & Martinez, J. Unexpected Reactivity of N-Acyl-Benzotriazoles with Aromatic Amines in Acidic Medium (ABAA Reaction). *Eur J Org Chem* **2019**, 85-90, doi:10.1002/ejoc.201801567 (2019).

3 van Herpt, J. T., Stuart, M. C., Browne, W. R. & Feringa, B. L. A dithienylethene-based rewritable hydrogelator. *Chemistry* **20**, 3077-3083, doi:10.1002/chem.201304064 (2014).

4 Lin, M. H. *et al.* Disulfiram can inhibit MERS and SARS coronavirus papain-like proteases via different modes. *Antiviral Res* **150**, 155-163, doi:10.1016/j.antiviral.2017.12.015 (2018).

5 Ghosh, A. K. *et al.* Severe acute respiratory syndrome coronavirus papain-like novel protease inhibitors: design, synthesis, protein-ligand X-ray structure and biological evaluation. *J Med Chem* **53**, 4968-4979, doi:10.1021/jm1004489 (2010).

6 Zhang, W. *et al.* Potent and selective inhibition of pathogenic viruses by engineered ubiquitin variants. *PLoS Pathog* **13**, e1006372, doi:10.1371/journal.ppat.1006372 (2017).

7 Lee, H. *et al.* Inhibitor recognition specificity of MERS-CoV papain-like protease may differ from that of SARS-CoV. *ACS Chem Biol* **10**, 1456-1465, doi:10.1021/cb500917m (2015).

8 Wang, L., Hu, W. & Fan, C. Structural and biochemical characterization of SADS-CoV papain-like protease 2. *Protein science : a publication of the Protein Society* **29**, 1228-1241, doi:10.1002/pro.3857 (2020).
