# Supplementary material for: Structure of papain-like protease from SARS-CoV-2 and its complexes with non-covalent inhibitors
Source: Nat Commun. 2021 Feb 2;12:743. doi: 10.1038/s41467-021-21060-3 (PMC7854729; doi:10.1038/s41467-021-21060-3)
Supplement: Supplementary file 3 — Description of Additional Supplementary Files [file 41467_2021_21060_MOESM3_ESM.pdf]

Description of Additional Supplementary Files

Supplementary Data 1: NMR spectra of compounds synthesized for studies conducted on SARS-CoV-2 PLpro.
