# Supplementary figures and images for: Structure of papain-like protease from SARS-CoV-2 and its complexes with non-covalent inhibitors
Source: Nat Commun. 2021 Feb 2;12:743. doi: 10.1038/s41467-021-21060-3 (PMC7854729; doi:10.1038/s41467-021-21060-3)

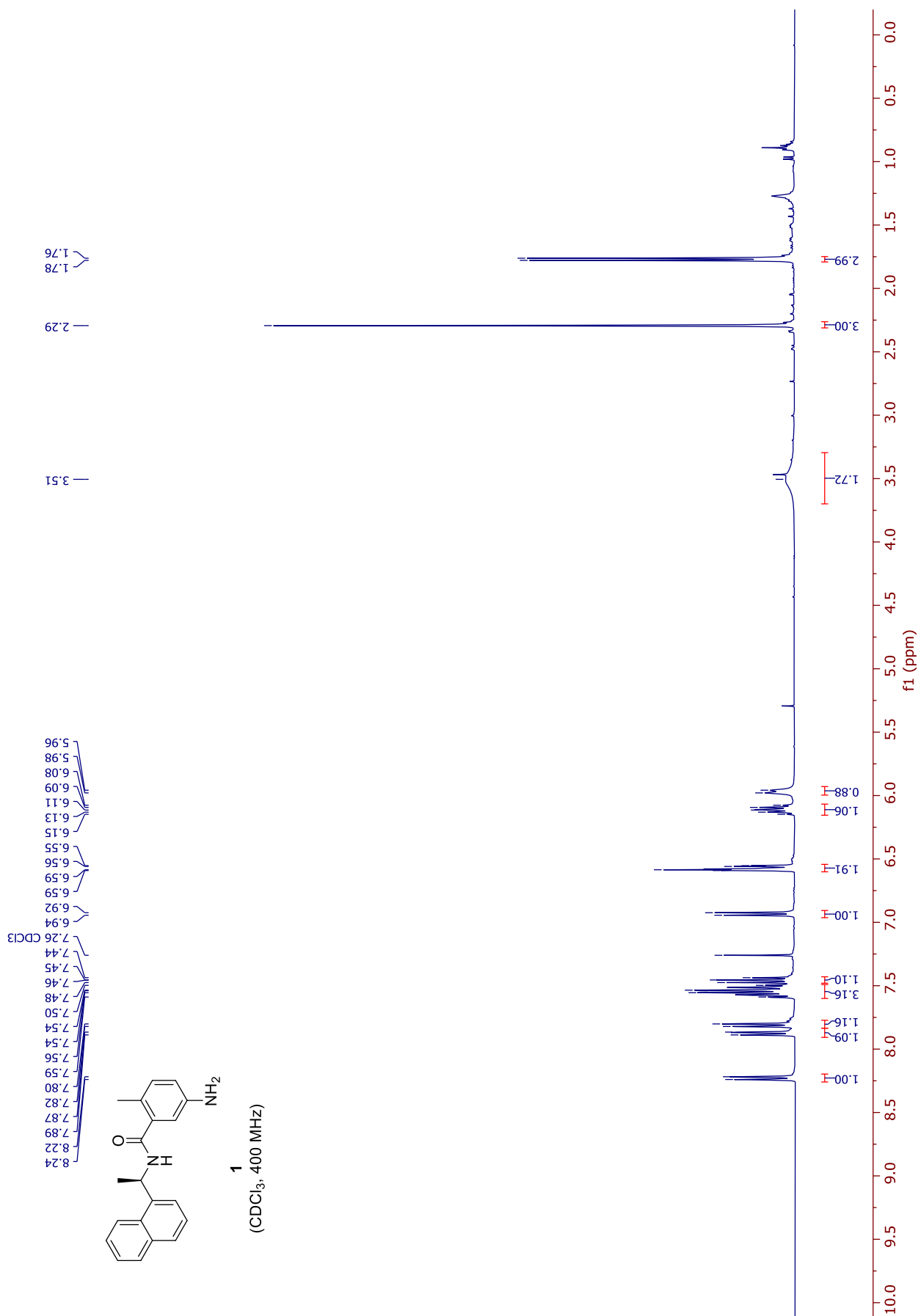

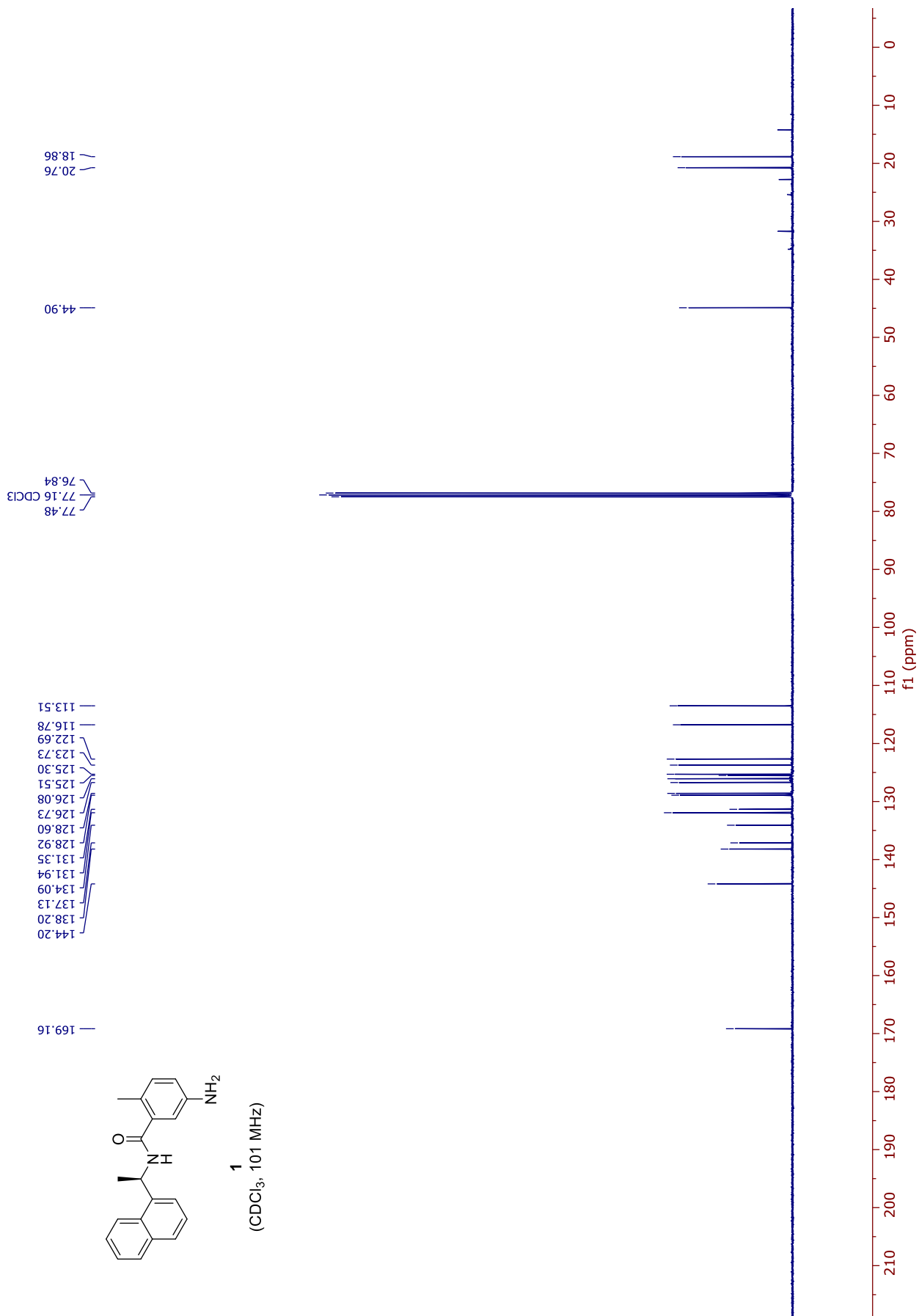

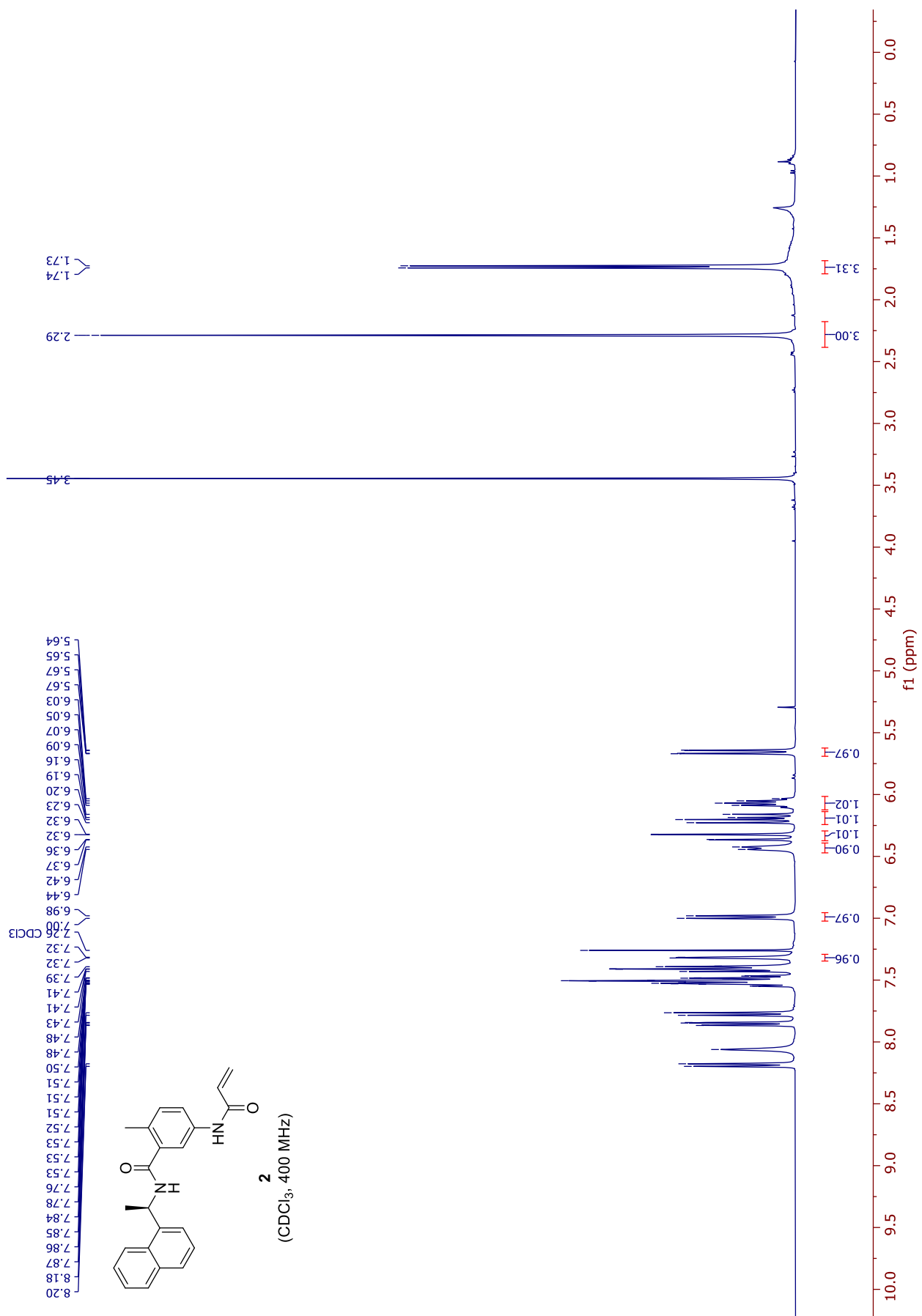

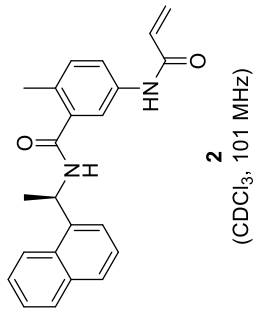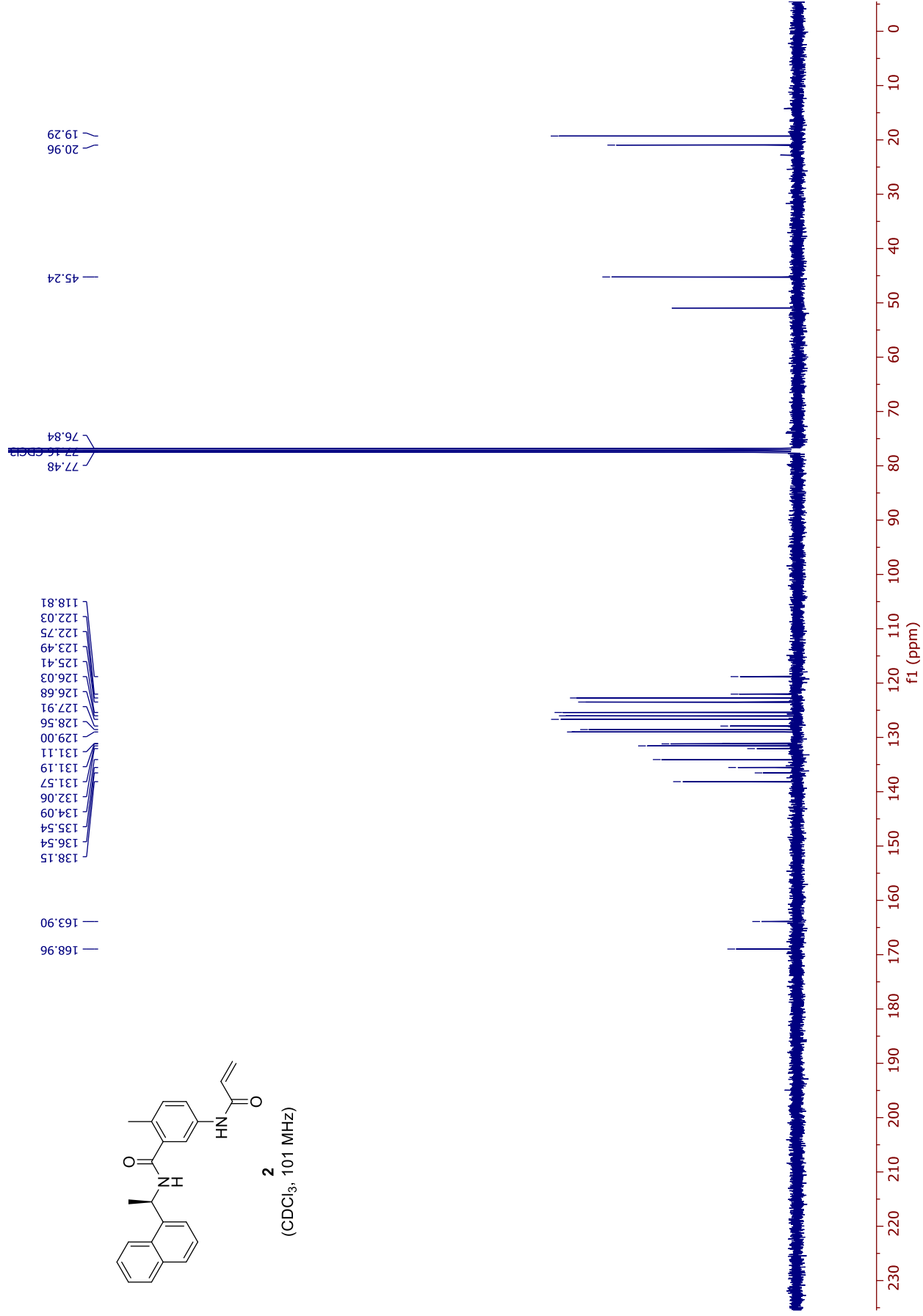

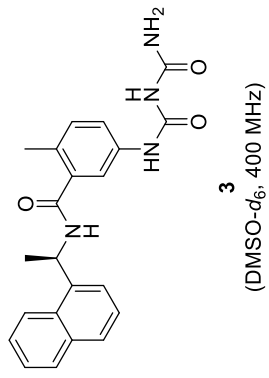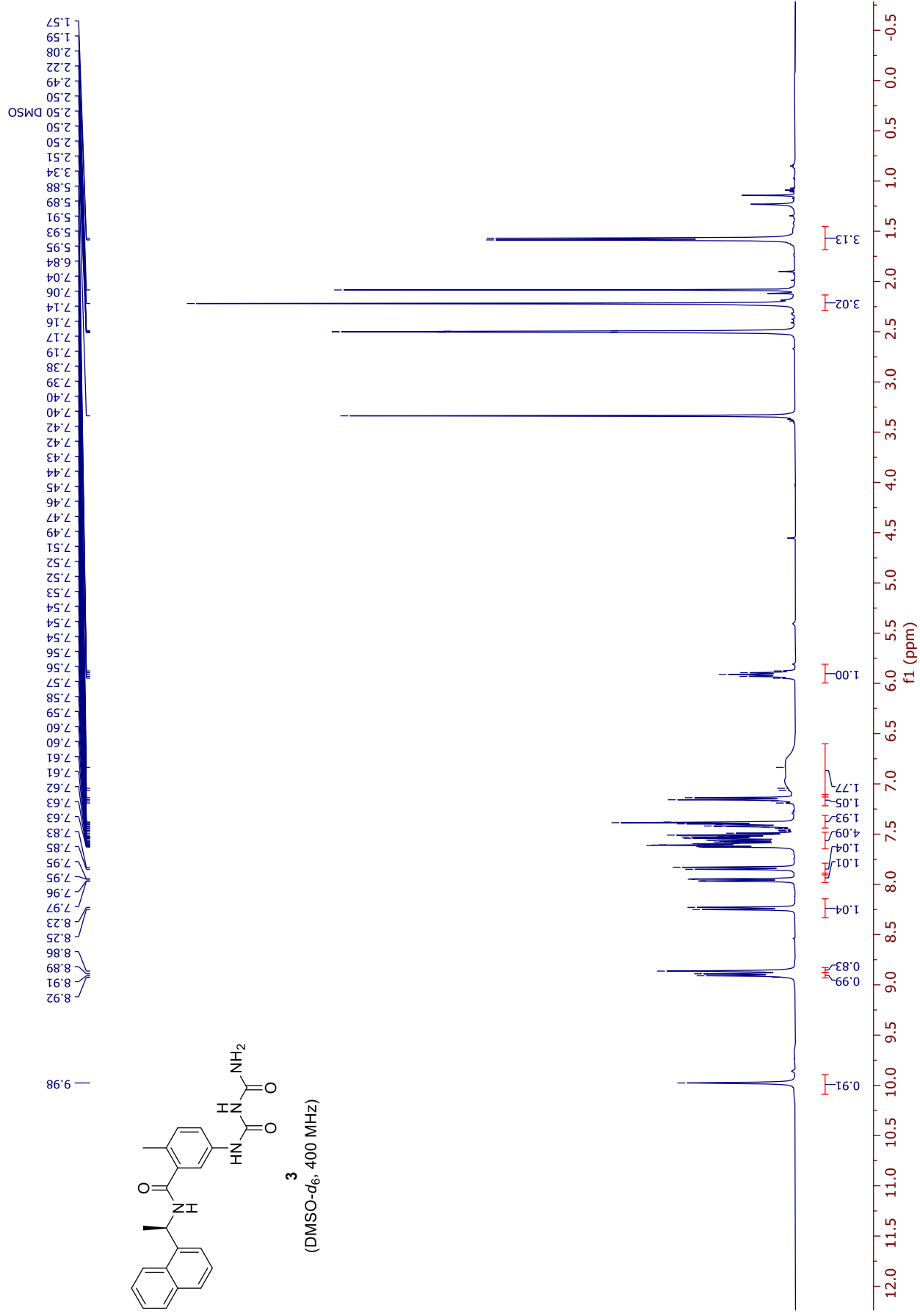

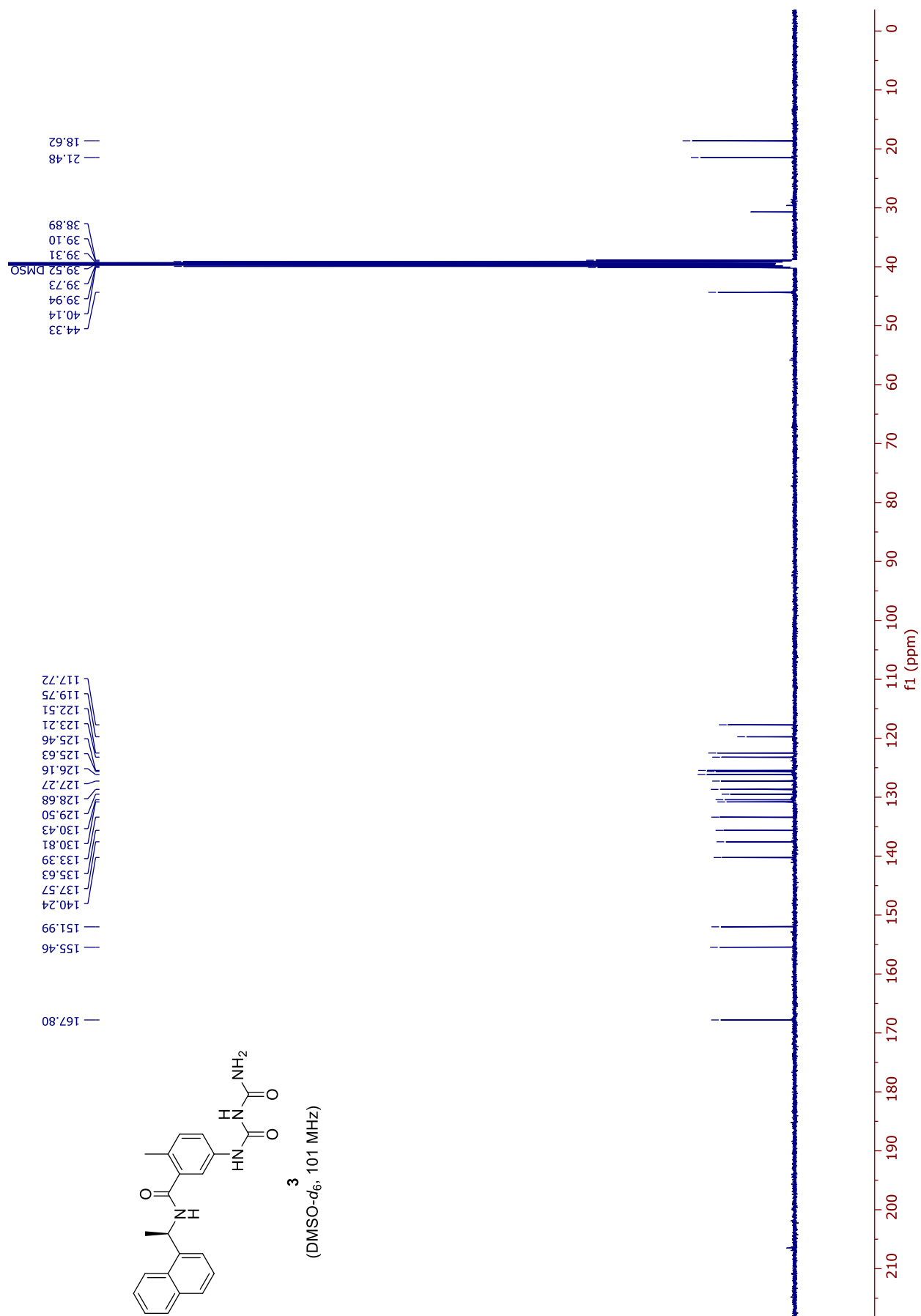

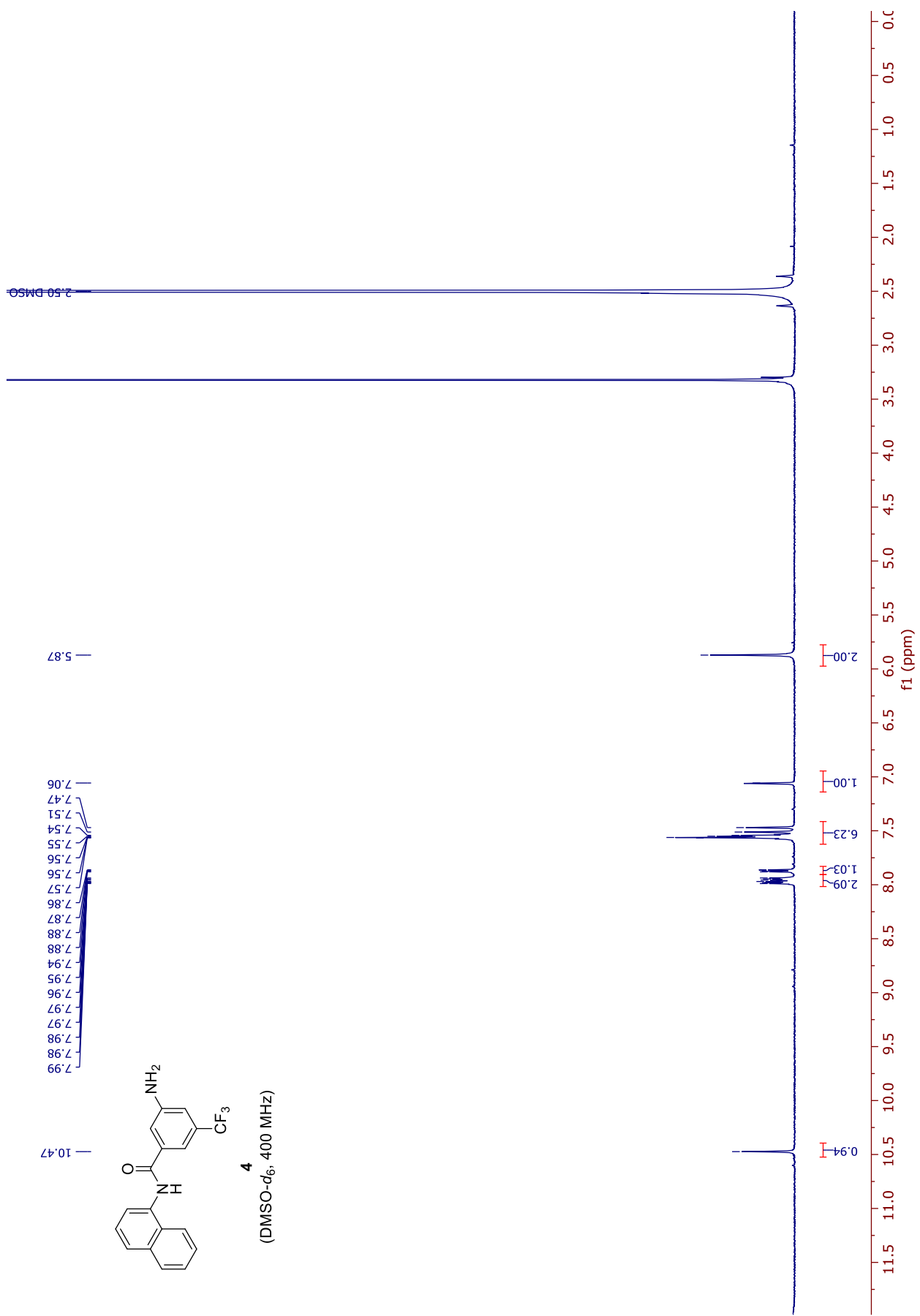

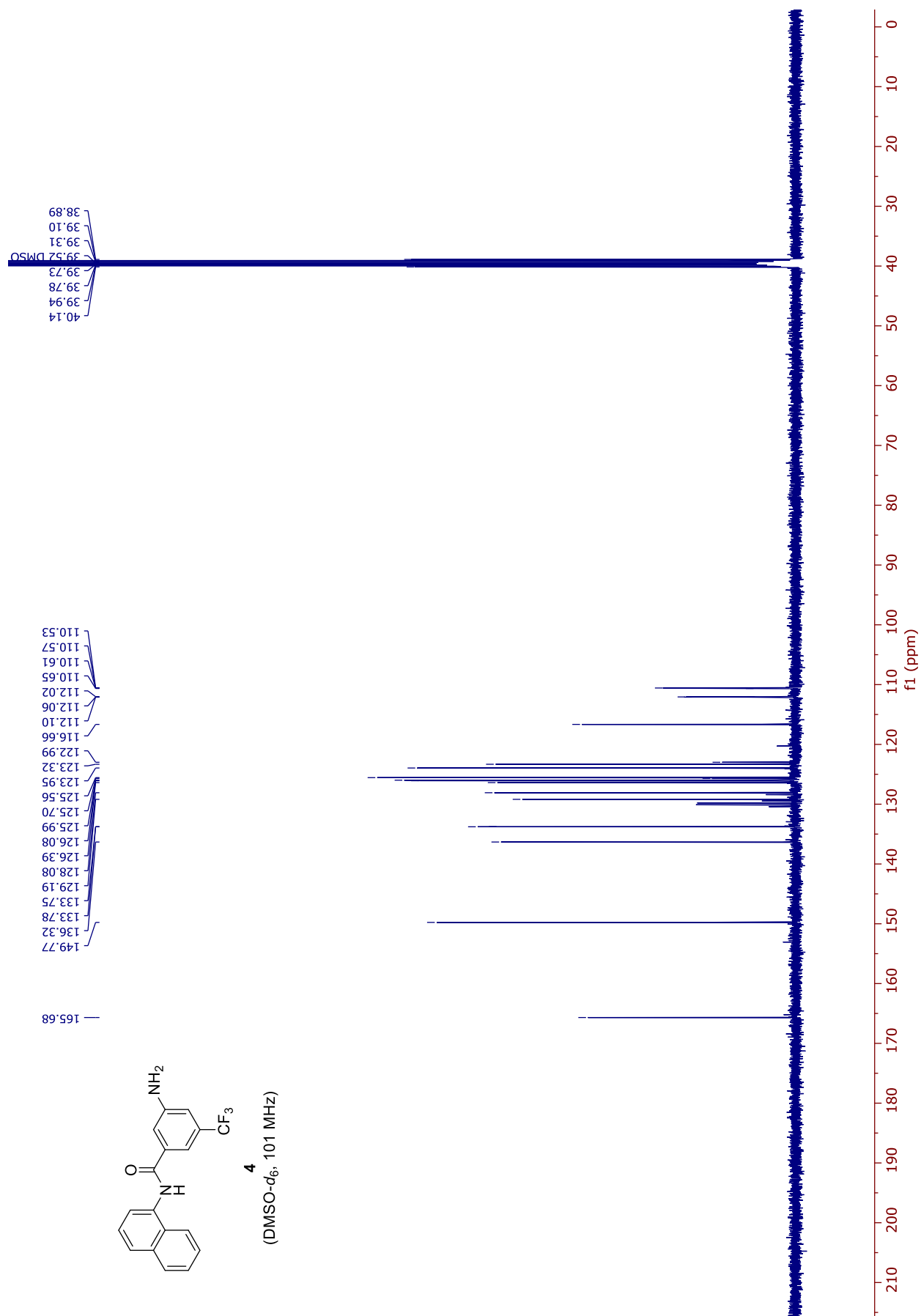

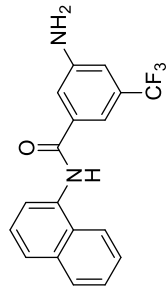

**4**

(DMSO- $d_6$ , 470 MHz)

— -61.34

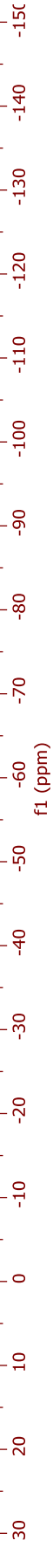

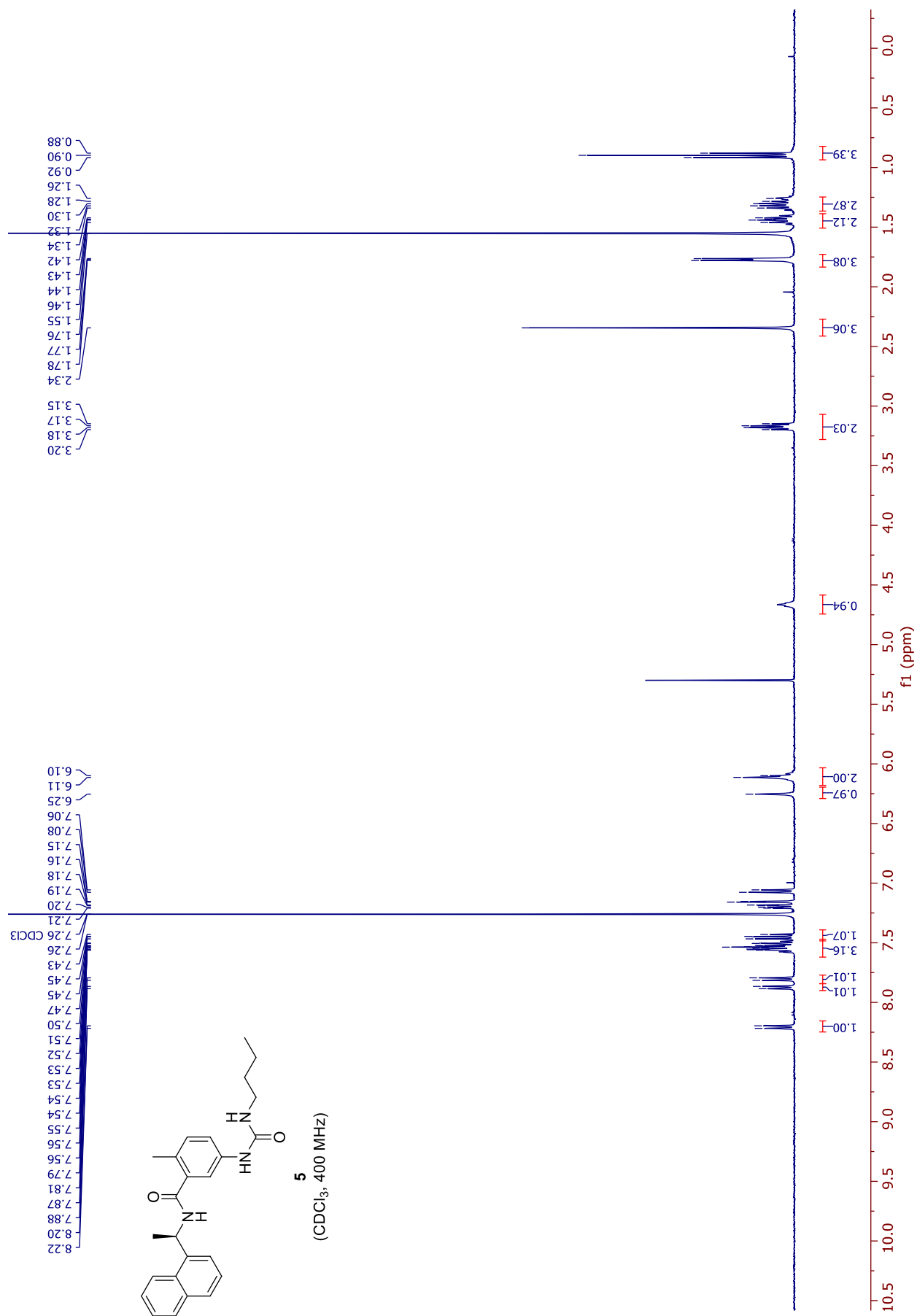

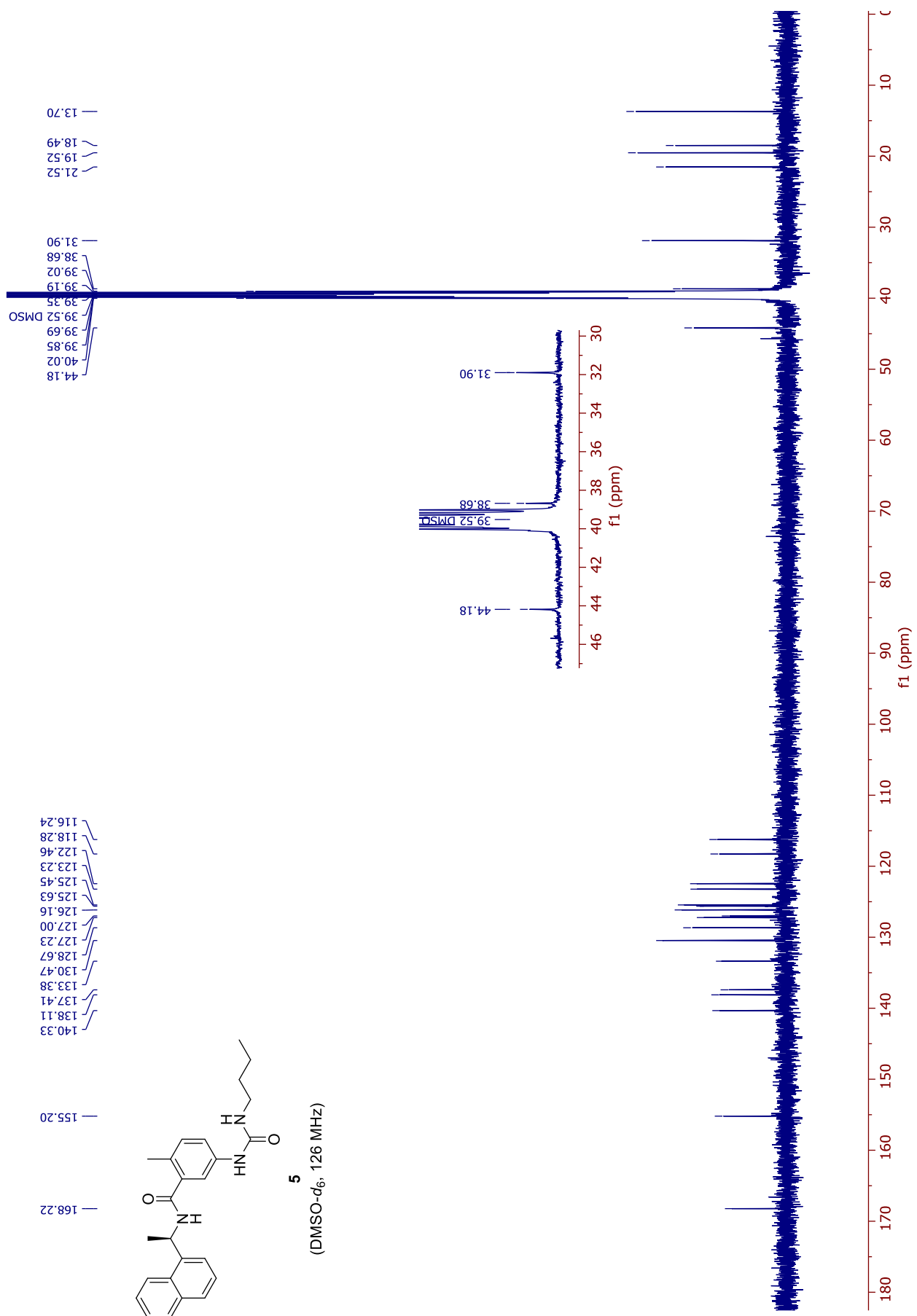

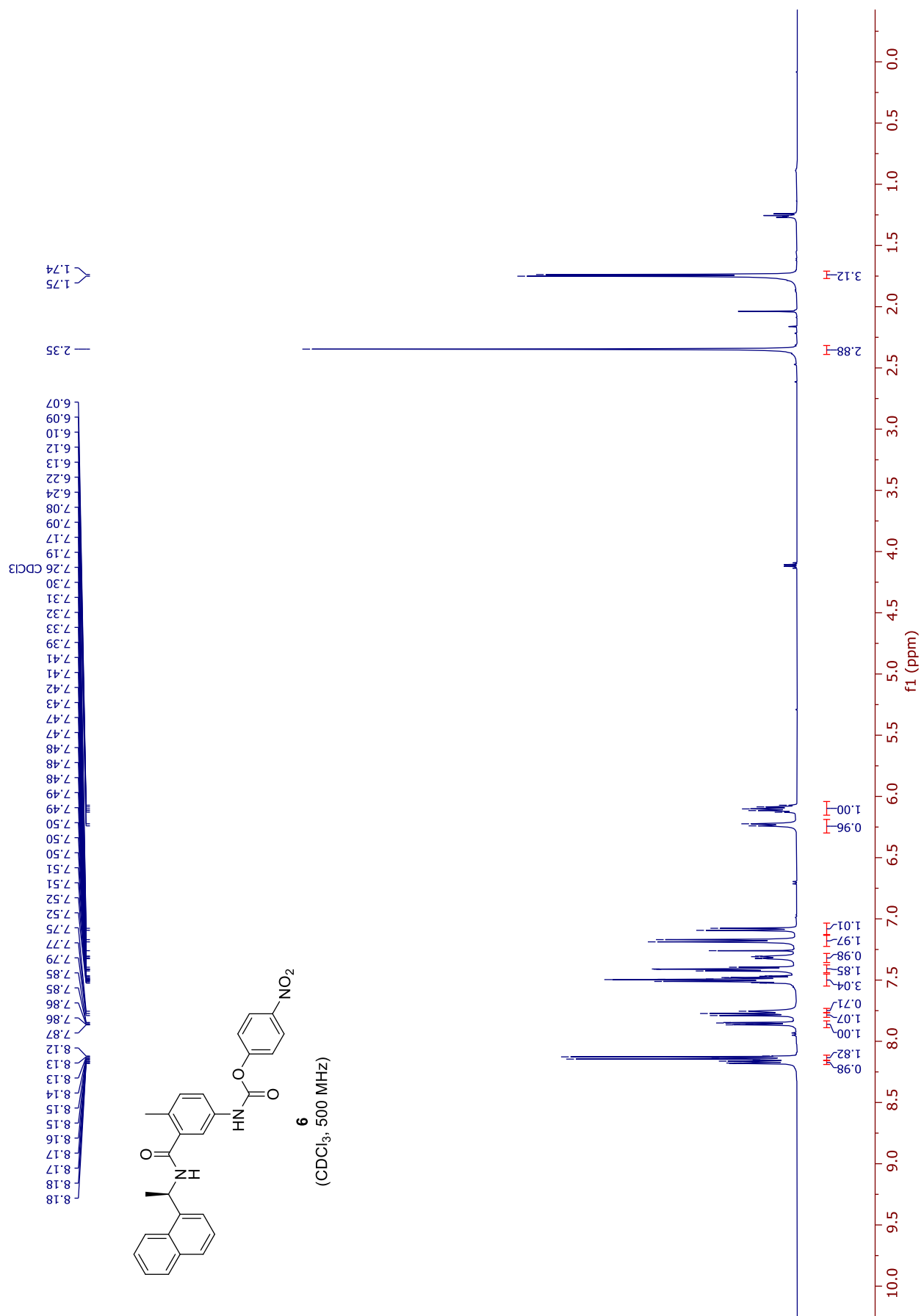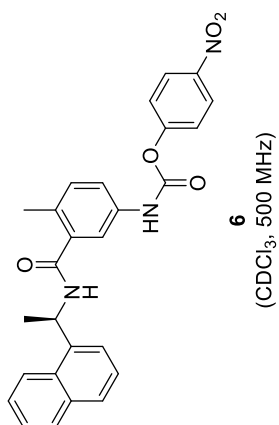

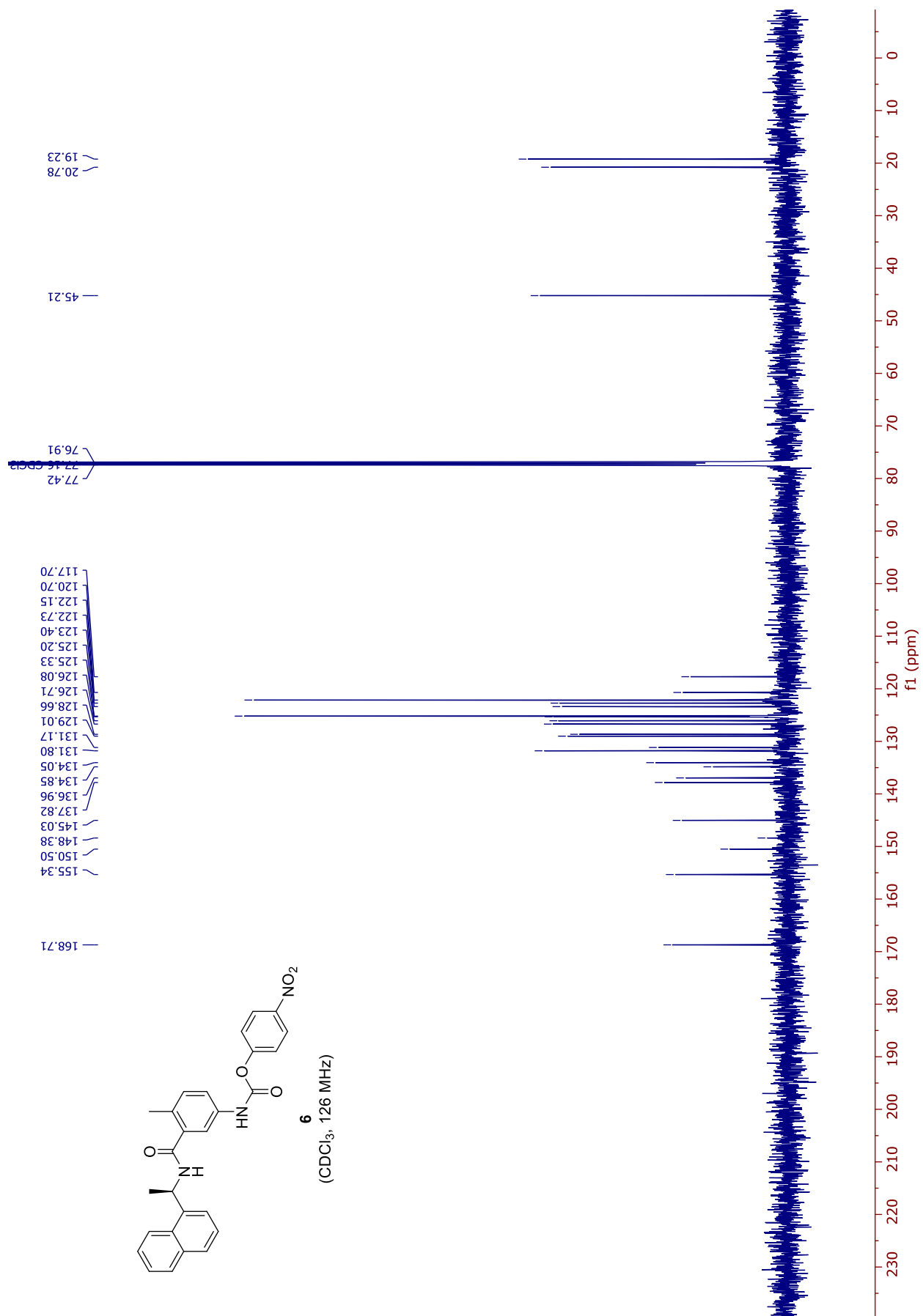

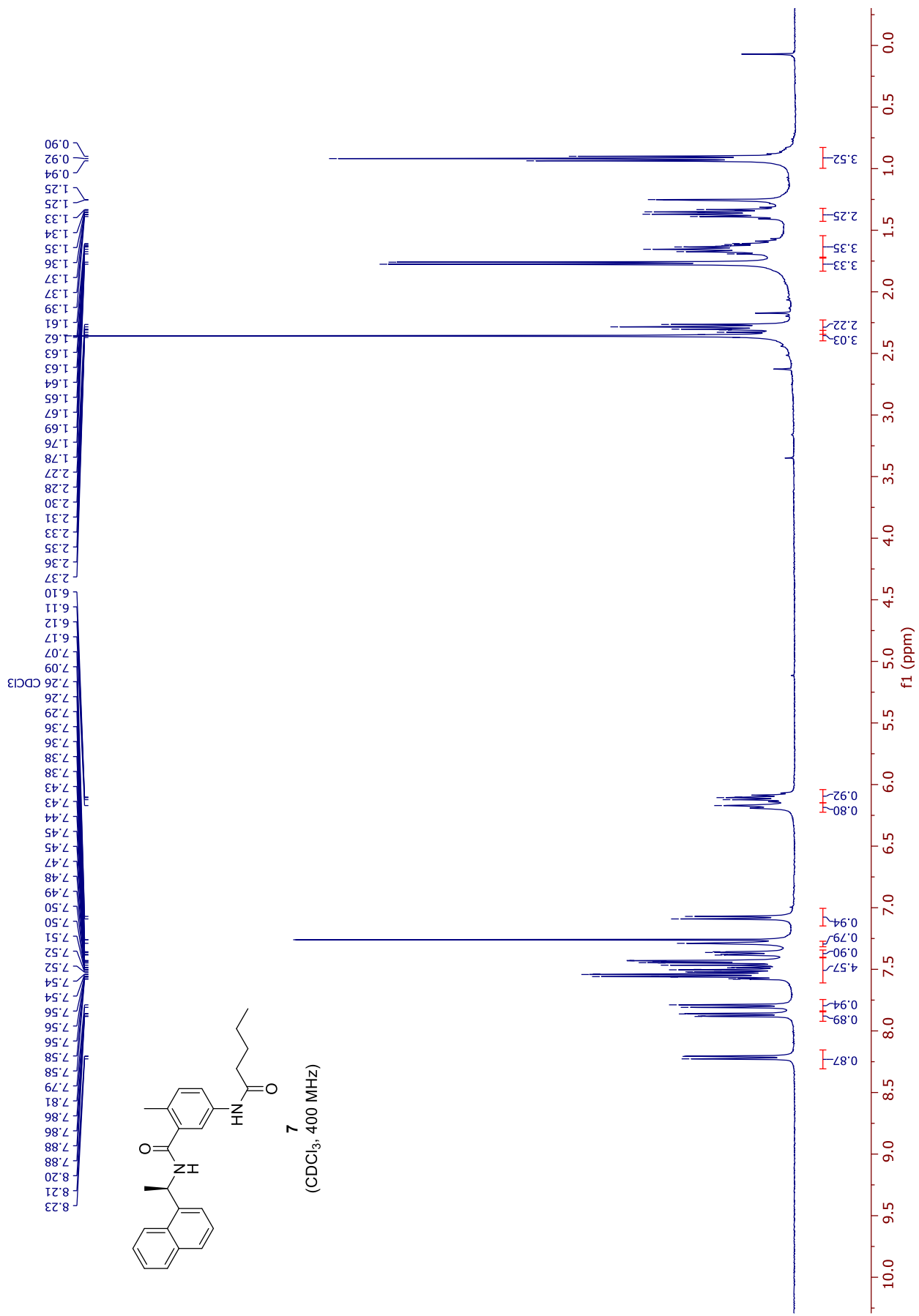

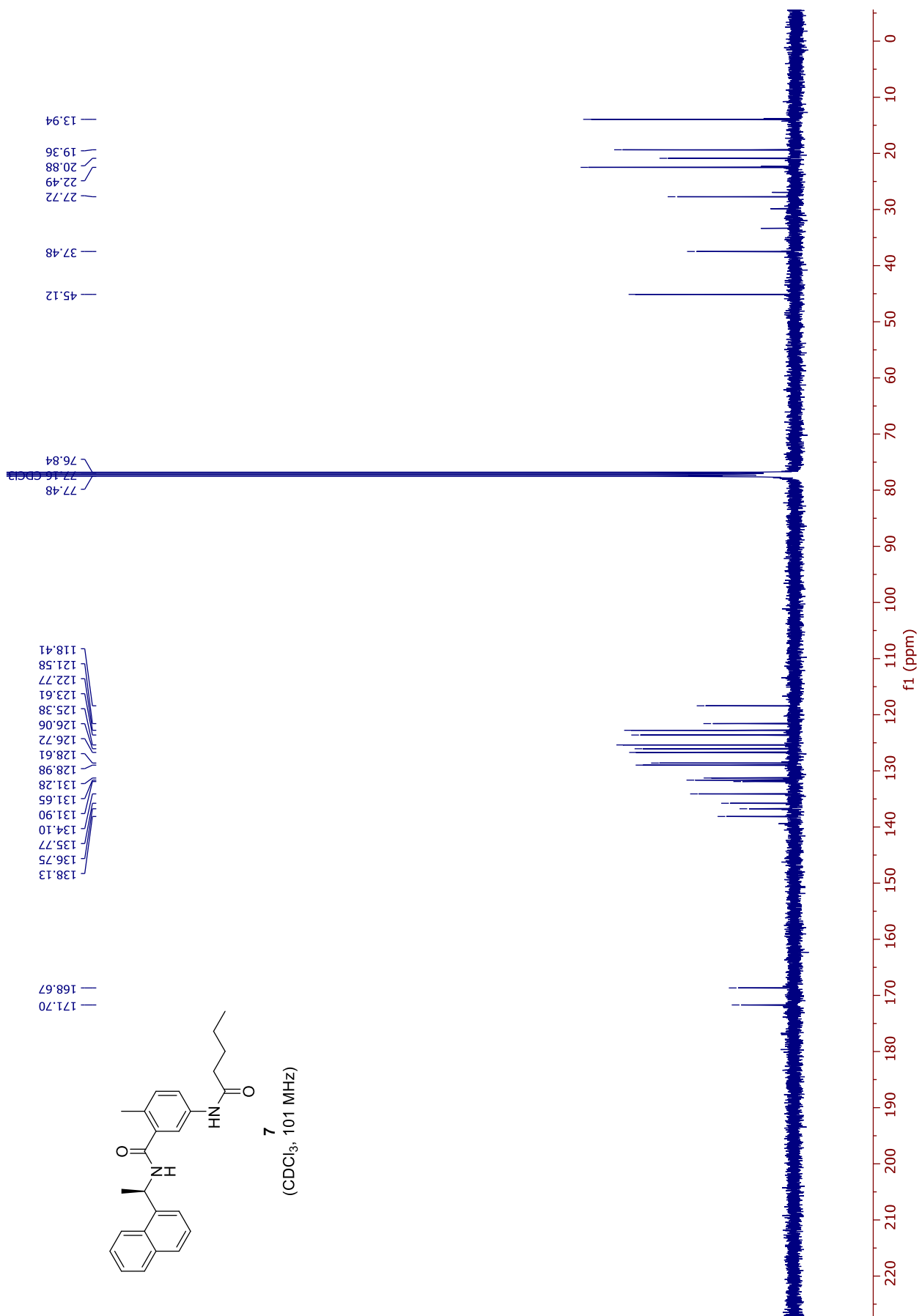

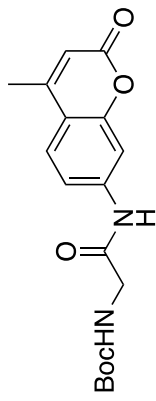

**S1**  
 $(^1\text{H NMR, (CD}_3\text{)}_2\text{SO, 400 MHz)}$

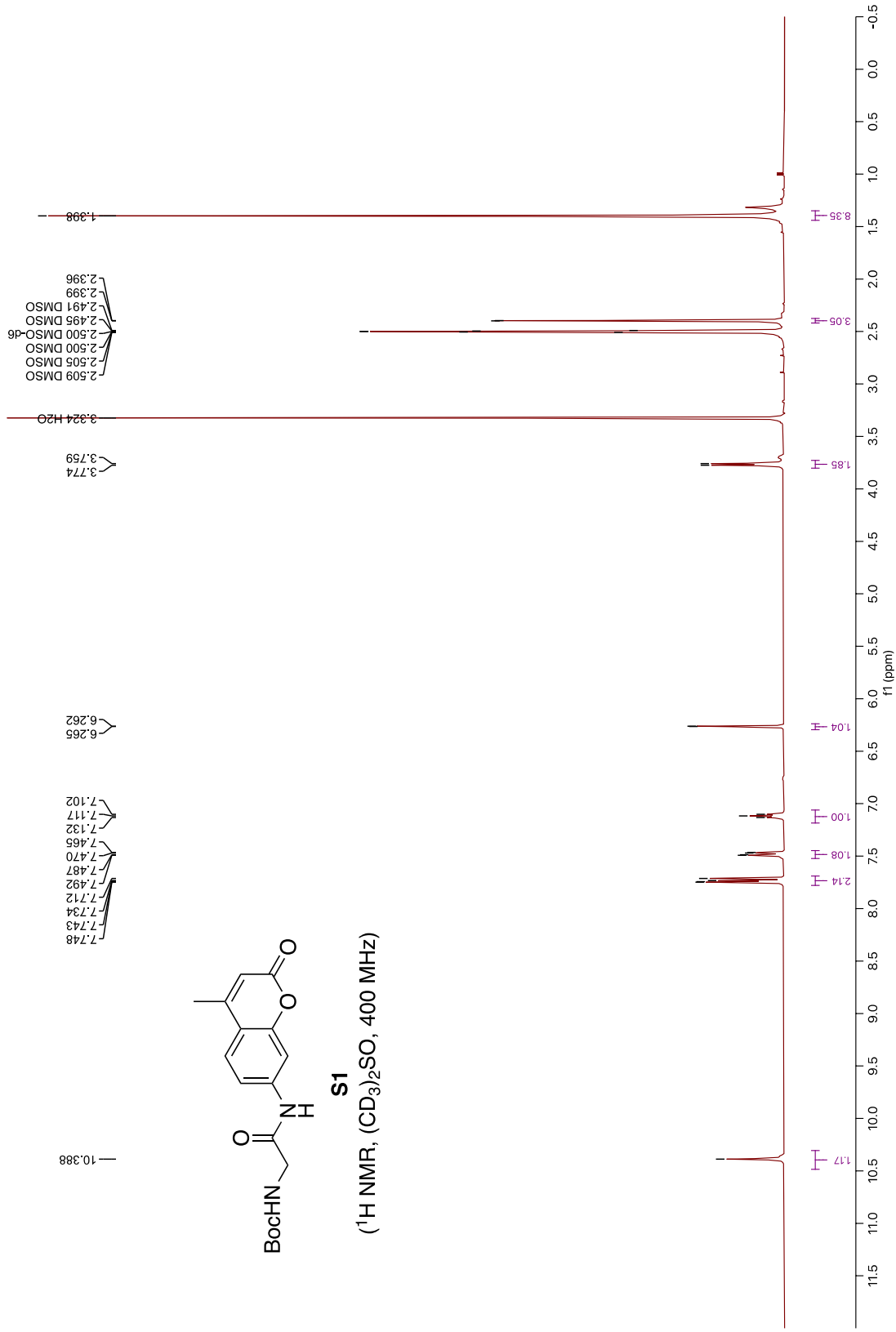

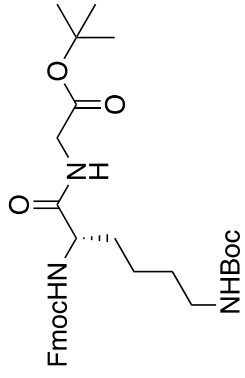

**S2**  
(<sup>1</sup>H NMR, CDCl<sub>3</sub>, 400 MHz)

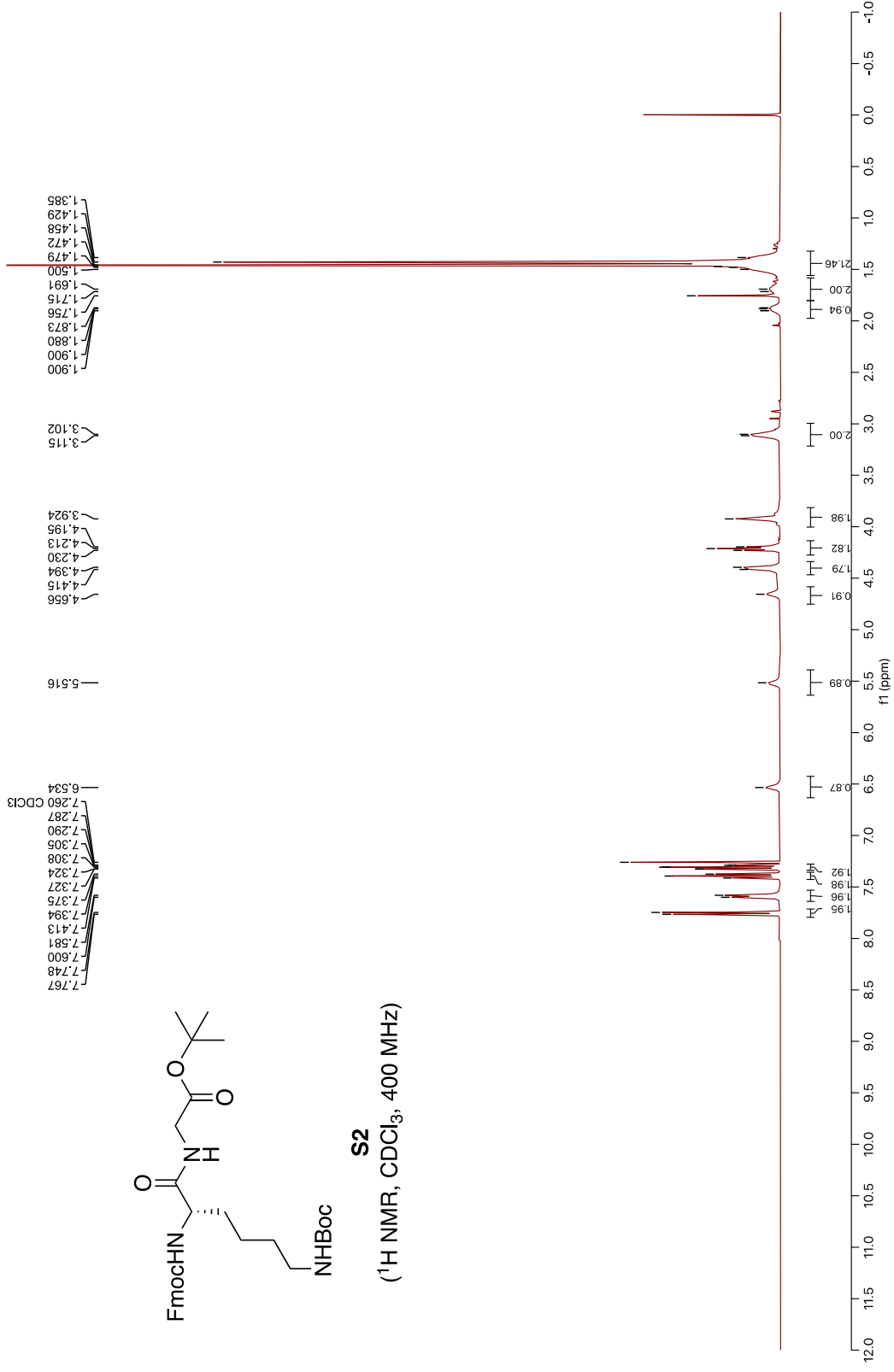

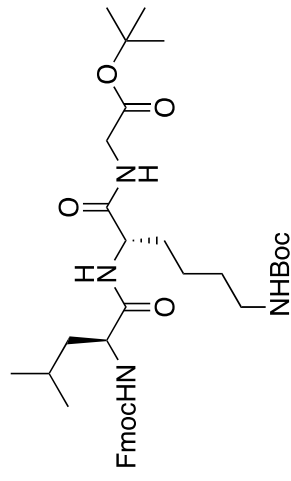

**S3**  
 $^1\text{H}$  NMR,  $\text{CDCl}_3$ , 400 MHz)

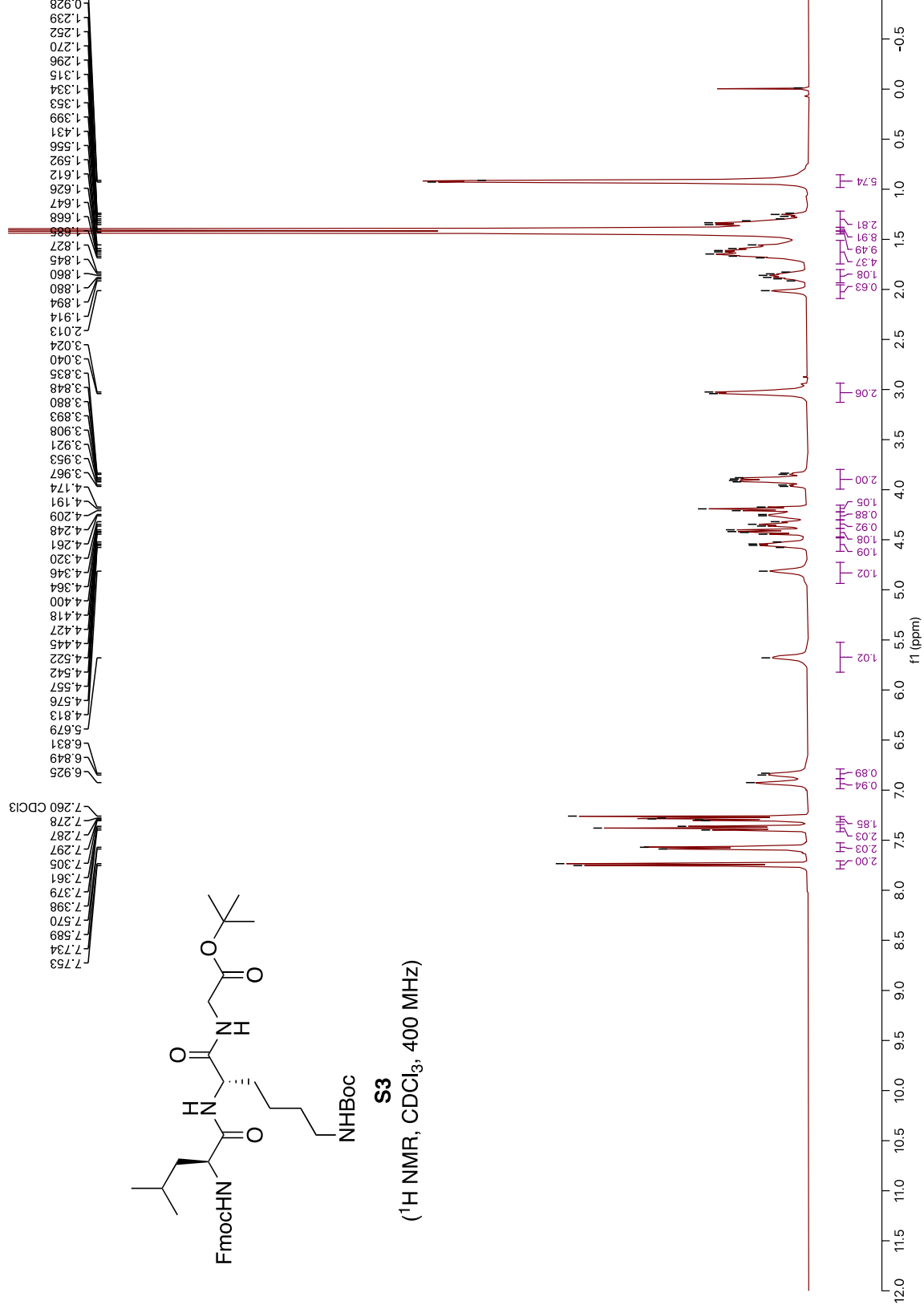

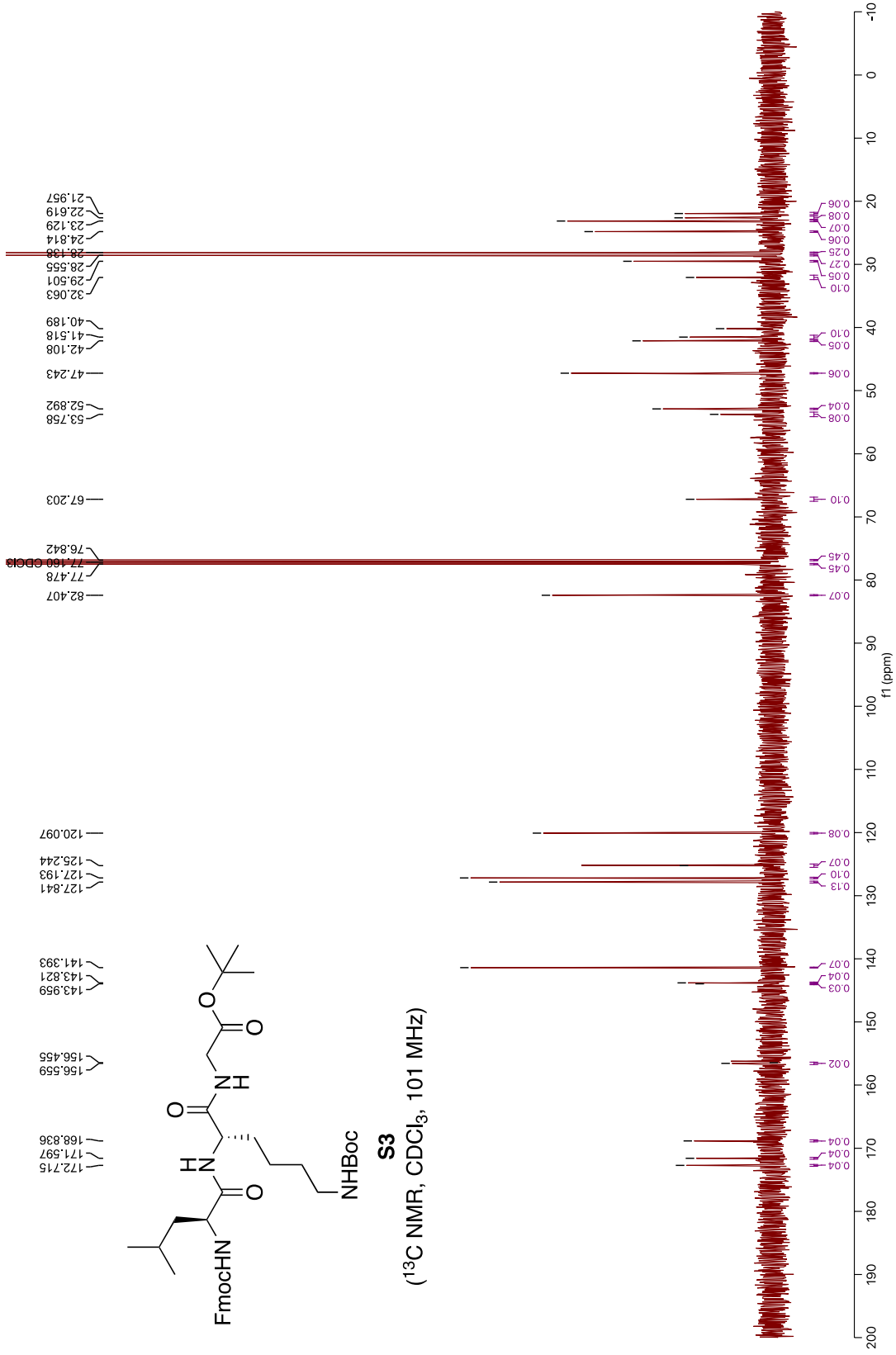

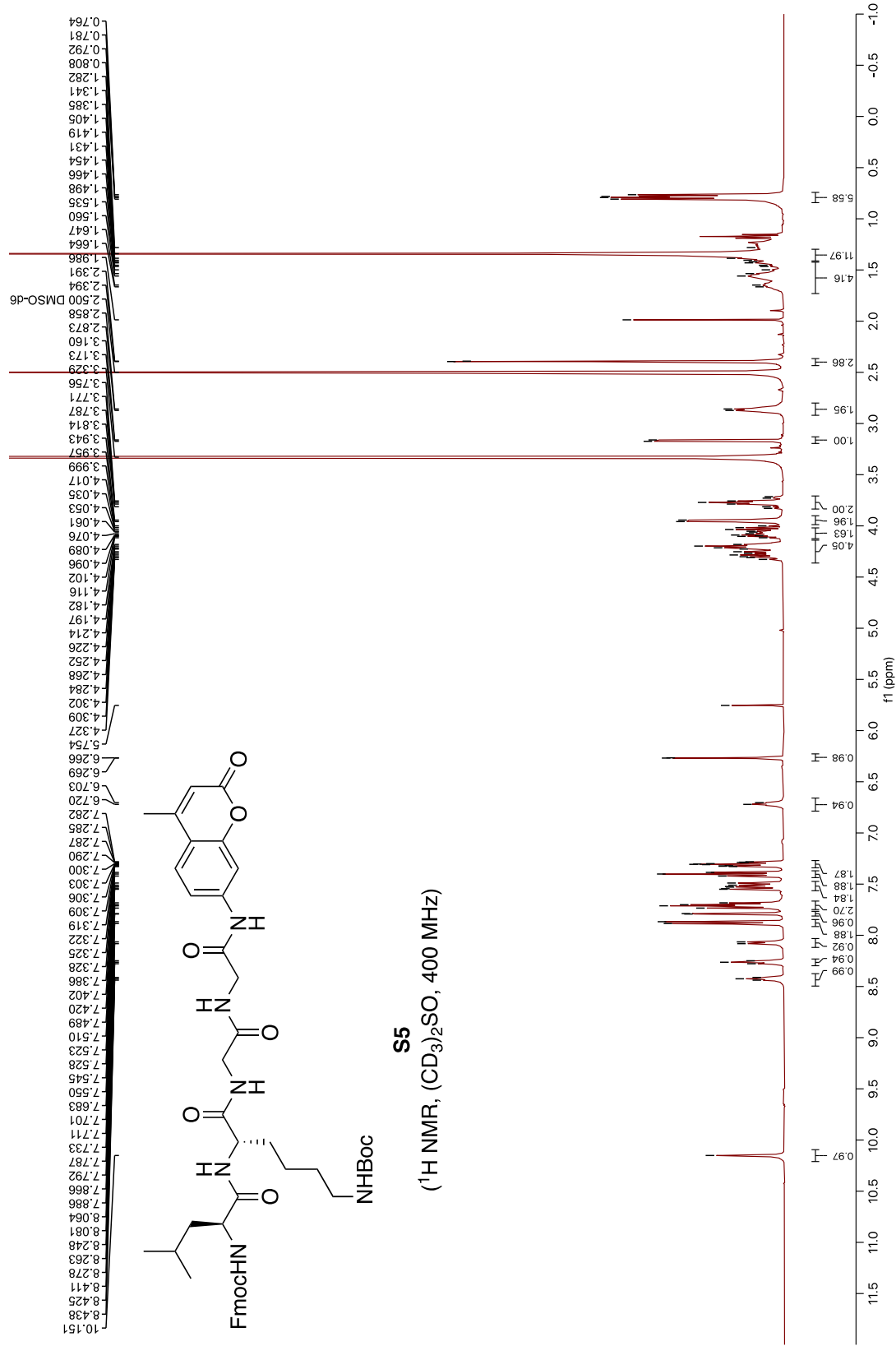

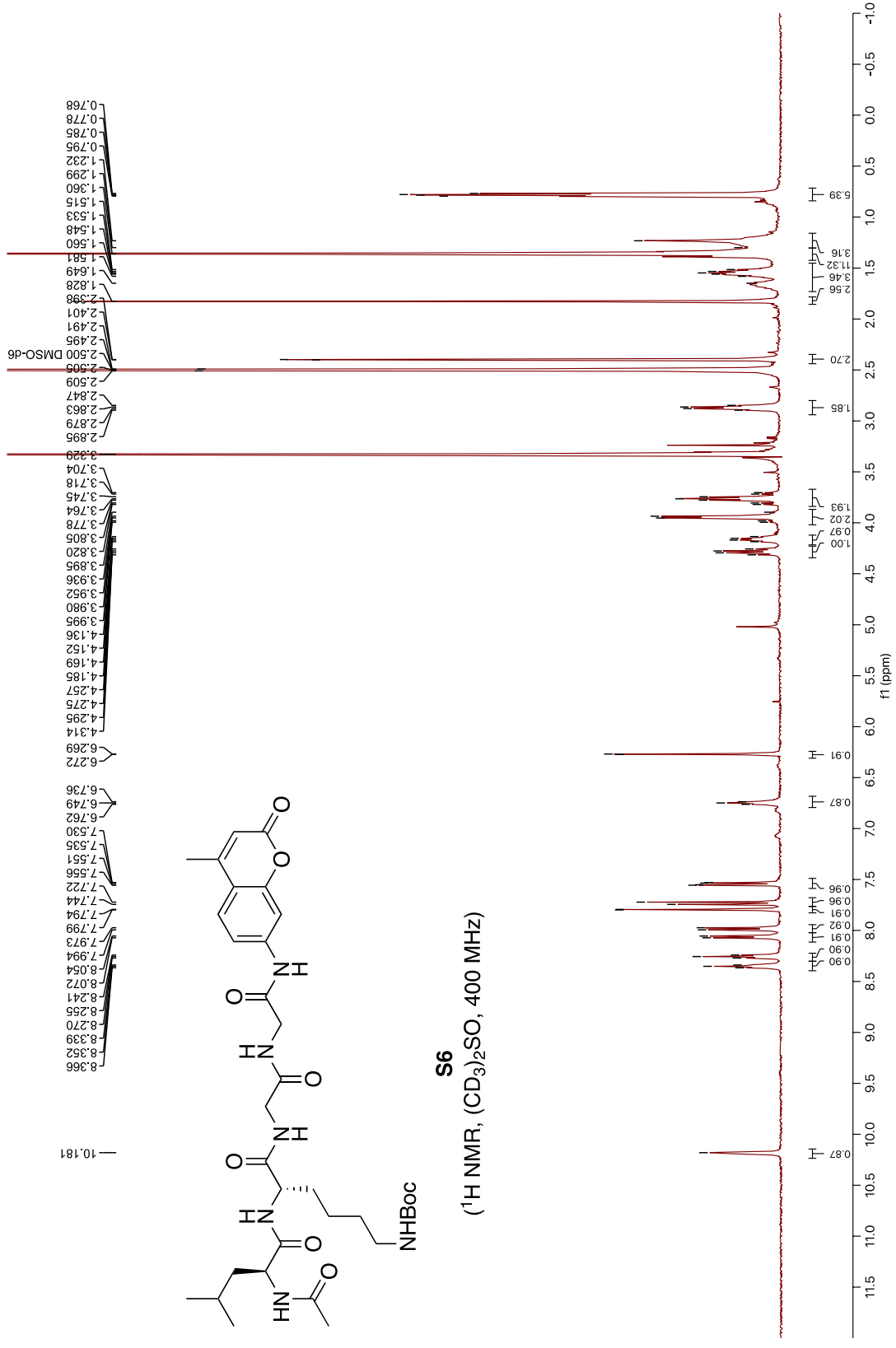

Supplement: Supplementary file 4 — Supplementary Data 1 [file 41467_2021_21060_MOESM4_ESM.pdf]
