# Supplementary material for: Structure of papain-like protease from SARS-CoV-2 and its complexes with non-covalent inhibitors
Source: Nat Commun. 2021 Feb 2;12:743. doi: 10.1038/s41467-021-21060-3 (PMC7854729; doi:10.1038/s41467-021-21060-3)
Supplement: Supplementary file 5 — Reporting summary [file 41467_2021_21060_MOESM5_ESM.pdf]

nature research

Corresponding author(s): Andrzej Joachimiak
Scott A. Snyder
Bryan C. Dickinson

Last updated by author(s): Dec. 8, 2020

Reporting Summary

Nature Research wishes to improve the reproducibility of the work that we publish. This form provides structure for consistency and transparency in reporting. For further information on Nature Research policies, see our Editorial Policies and the Editorial Policy Checklist.

Statistics

For all statistical analyses, confirm that the following items are present in the figure legend, table legend, main text, or Methods section.

n/a	Confirmed	
☐	☒	The exact sample size (*n*) for each experimental group/condition, given as a discrete number and unit of measurement
☒	☐	A statement on whether measurements were taken from distinct samples or whether the same sample was measured repeatedly
☒	☐	The statistical test(s) used AND whether they are one- or two-sided<br>*Only common tests should be described solely by name; describe more complex techniques in the Methods section.*
☒	☐	A description of all covariates tested
☒	☐	A description of any assumptions or corrections, such as tests of normality and adjustment for multiple comparisons
☐	☒	A full description of the statistical parameters including central tendency (e.g. means) or other basic estimates (e.g. regression coefficient) AND variation (e.g. standard deviation) or associated estimates of uncertainty (e.g. confidence intervals)
☒	☐	For null hypothesis testing, the test statistic (e.g. *F*, *t*, *r*) with confidence intervals, effect sizes, degrees of freedom and *P* value noted<br>*Give P values as exact values whenever suitable.*
☒	☐	For Bayesian analysis, information on the choice of priors and Markov chain Monte Carlo settings
☒	☐	For hierarchical and complex designs, identification of the appropriate level for tests and full reporting of outcomes
☒	☐	Estimates of effect sizes (e.g. Cohen's *d*, Pearson's *r*), indicating how they were calculated

Our web collection on statistics for biologists contains articles on many of the points above.

Software and code

Policy information about availability of computer code

Data collection	The x-ray diffraction experiments were collected using SBCcollect software (ver2 0.9.0.3 ID Revision: 32) on the PILATUS3 X 6M detector.
Data analysis	The data were integrated and scaled with the HKL3000 suite v720. Intensities were converted to structure factor amplitudes in the Ctruncate v1.17.29 program from the CCP4 v7.1.000 package. The structures were determined using MOLREP v11.7.02 implemented in the HKL3000 v720 software package. The initial solutions were refined by REFMAC v5.8.0258 as a part of HKL3000. The models including the ligands were adjusted using COOT v0.7.2 and then iteratively refined using COOT and PHENIX (v1.17.1-3660 for apo structures and v1.18.2-3874 for ligand structures). The stereochemistry of the structure was validated with PHENIX suite (v1.17.1-3660) incorporating MOLPROBITY v 4.02b-467 tools and validated with the Protein Data Bank validation pipeline v2.13.1. PLpro inhibition data were fit using nonlinear regression (dose-response inhibition, variable slope) analysis in GraphPad Prism 7.0

For manuscripts utilizing custom algorithms or software that are central to the research but not yet described in published literature, software must be made available to editors and reviewers. We strongly encourage code deposition in a community repository (e.g. GitHub). See the Nature Research guidelines for submitting code & software for further information.

Data

Policy information about availability of data

All manuscripts must include a data availability statement. This statement should provide the following information, where applicable:

- Accession codes, unique identifiers, or web links for publicly available datasets
- A list of figures that have associated raw data
- A description of any restrictions on data availability

The structural datasets generated during the current study are available in the Protein Data Bank repository (https://www.rcsb.org/) under accession codes: 6WZU, 6WRH, 6XG3, 7JIR, 7JIT, 7JIV and 7JIW. Diffraction images are available on server in Dr. W. Minor laboratory https://proteindiffraction.org. Plasmid for expression

PLpro (NR-52897 Vector pMCSG53 Containing the SARS-Related Coronavirus 2, Wuhan-Hu-1 Papain-Like Protease) is available in the NIH the BEI Resources Repository (https://www.niaid.nih.gov/research/bei-resources-repository). All other data generated during the current study including the raw kinetic and biophysical data are available upon request.

Field-specific reporting

Please select the one below that is the best fit for your research. If you are not sure, read the appropriate sections before making your selection.

☒ Life sciences ☐ Behavioural & social sciences ☐ Ecological, evolutionary & environmental sciences

For a reference copy of the document with all sections, see nature.com/documents/nr-reporting-summary-flat.pdf

Life sciences study design

All studies must disclose on these points even when the disclosure is negative.

Sample size	PLpro protease from SARS CoV-2 was purified multiple times, samples show very similar purity, enzyme activity and crystallization properties
Data exclusions	No exclusions
Replication	Seven crystal structures were determined, standard protocols were used to assure reproducibility, different protein preparation produced this same quality of crystals
Randomization	Throughout the refinement, the same 5% of randomly selected reflections were kept out throughout from the refinement (in both REFMAC and PHENIX refinement) to assess quality.
Blinding	Randomly selected set of reflection serves as internal standard of refinement and model quality

Reporting for specific materials, systems and methods

We require information from authors about some types of materials, experimental systems and methods used in many studies. Here, indicate whether each material, system or method listed is relevant to your study. If you are not sure if a list item applies to your research, read the appropriate section before selecting a response.

Materials & experimental systems

n/a	Involved in the study	
☐	☒	Antibodies
☐	☒	Eukaryotic cell lines
☒	☐	Palaeontology and archaeology
☐	☒	Animals and other organisms
☒	☐	Human research participants
☒	☐	Clinical data
☒	☐	Dual use research of concern

Methods

n/a	Involved in the study	
☒	☐	ChIP-seq
☒	☐	Flow cytometry
☒	☐	MRI-based neuroimaging

Antibodies

Antibodies used	Antibodies used: monoclonal mouse-anti-SARS-CoV-2 spike antibody [1A9] Catalog number GTX632604, GeneTex and secondary antibody (ImmPRESS Horse Anti-Mouse IgG Polymer Reagent, Peroxidase, catalog number MP-7402-50; Vector Laboratories
Validation	Monoclonal mouse-anti-SARS-CoV-2 spike antibody [1A9] Catalog number GTX632604, GeneTex was validated for application with WB, ICC/IF, IHC-P, FACS, IP, ELISA, Sandwich ELISA, IHC-P (cell pellet)<br>ImmPRESS Horse Anti-Mouse IgG Polymer Reagent, Peroxidase, catalog number MP-7402-50; Vector Laboratorie was validated for applications in mmunohistochemistry / immunocytochemistry, in situ hybridization and ELISAs

Eukaryotic cell lines

Policy information about cell lines

Cell line source(s)	Vero E6 cells - VERO C1008 [Vero 76, clone E6, Vero E6] (ATCC® CRL-1586™)
Authentication	None of cell lines were authenticated
Mycoplasma contamination	As tested by vendor

Commonly misidentified lines (See ICLAC register)	*Name any commonly misidentified cell lines used in the study and provide a rationale for their use.*

Animals and other organisms

Policy information about studies involving animals; ARRIVE guidelines recommended for reporting animal research

Laboratory animals	Escherichia coli
Wild animals	*Provide details on animals observed in or captured in the field; report species, sex and age where possible. Describe how animals were caught and transported and what happened to captive animals after the study (if killed, explain why and describe method; if released, say where and when) OR state that the study did not involve wild animals.*
Field-collected samples	*For laboratory work with field-collected samples, describe all relevant parameters such as housing, maintenance, temperature, photoperiod and end-of-experiment protocol OR state that the study did not involve samples collected from the field.*
Ethics oversight	*Identify the organization(s) that approved or provided guidance on the study protocol, OR state that no ethical approval or guidance was required and explain why not.*

Note that full information on the approval of the study protocol must also be provided in the manuscript.
